# Optimization of Pulsed Saturation Transfer MR Fingerprinting (ST MRF) Acquisition Using the Cramér–Rao Bound and Sequential Quadratic Programming

**DOI:** 10.1002/mrm.70141

**Published:** 2025-10-18

**Authors:** Nikita Vladimirov, Moritz Zaiss, Or Perlman

**Affiliations:** ^1^ School of Biomedical Engineering Tel Aviv University Tel Aviv Israel; ^2^ Institute of Neuroradiology University Hospital Erlangen, Friedrich‐Alexander‐Universität Erlangen‐Nürnberg (FAU) Erlangen Germany; ^3^ Department of Artificial Intelligence in Biomedical Engineering Friedrich‐Alexander‐Universität Erlangen‐Nürnberg (FAU) Erlangen Germany; ^4^ Sagol School of Neuroscience Tel Aviv University Tel Aviv Israel

**Keywords:** CEST, MR Fingerprinting, MT, optimization, quantitative MRI

## Abstract

**Purpose:**

To develop a method for optimizing pulsed saturation transfer MR fingerprinting (ST MRF) acquisition.

**Methods:**

The Cramér–Rao bound (CRB) for variance assessment was employed on Bloch–McConnell‐based simulated signals, followed by a numerical sequential quadratic programming optimization and basin‐hopping avoidance of local minima. Validation was performed using L‐arginine phantoms and healthy human volunteers at 3T while restricting the scan time to be less than 40 s.

**Results:**

The proposed optimization approach resulted in a significantly improved agreement with reference standard values in vivo, compared to baseline non‐optimized protocols (8% lower NRMSE, 7% higher SSIM, and 15% higher Pearson's r value, p<0.001).

**Conclusion:**

The combination of the CRB with sequential quadratic programming and a rapid Bloch–McConnell simulator offers a means for optimizing and accelerating pulsed CEST and semisolid magnetization transfer (MT) MRF acquisition.

## 1 Introduction

Chemical exchange saturation transfer (CEST) and semisolid magnetization transfer (MT) MRI leverage the saturation transfer (ST) mechanism to extract molecular information [1]. Over the years, these approaches have proven valuable for preclinical biological investigations and clinical human studies [2, 3, 4]. By informing on molecular events associated with altered metabolite composition, compound concentration, or pH, ST MRI provides a radiation‐free alternative to positron emission tomography (PET) and single photon emission computed tomography (SPECT) while offering improved spatial resolution compared to MR spectroscopic imaging (MRSI) [5, 6, 7].

ST MRI operates by applying selective radio‐frequency pulses to saturate exchangeable protons on mobile proteins, peptides, macromolecules, or metabolites, which then transfer this saturation to bulk water protons. The semisolid MT contrast is a known biomarker for myelin integrity in multiple sclerosis [8]. In addition, amide proton transfer (APT) has been employed to detect pH changes during stroke [9, 10] and to differentiate tumor progression from radiation necrosis in glioma patients [11, 12], while glutamate CEST has been used to characterize neurodegenerative, psychiatric, and oncological disease [13, 14, 15]. Many other ST‐based applications have been reported and are the subject of research. These include glycogen imaging [16], protein aggregation detection [17], reporter gene and liposome imaging [18, 19, 20], glucose uptake analysis [21, 22], and cardiac metabolism characterization [23].

While ST‐*weighted* imaging has demonstrated a marked potential, especially for brain tumors [24], the technique still faces several challenges that must be overcome before the full extent of this contrast mechanism can be exploited. A key limitation is the semi‐quantitative nature of CEST and semisolid MT imaging; the native ST signal not only contains the product of contributions from the proton volume fraction ($f_{s}$) and exchange rate ($k_{ex}$) of multiple compounds and metabolites, but is also affected by water T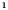 and the particular parameters of the pulse sequence used [25].

Quantitative imaging is a desirable outcome when developing MR methods, because it enables reproducibility and cross‐study comparisons while facilitating a physically meaningful interpretation of image data [26, 27, 28]. ST MRI is no exception, and accurate quantification of proton exchange parameters has been the goal of various previous research efforts [29, 30, 31]. Several foundational methods, such as quantifying exchange rates using the saturation time/saturation power (QUEST/QUESP) [2, 29, 32], Omega plots [33, 34], and Bloch–McConnell (BM) fitting [2, 35], rely on analytical models derived from the BM equations. These methods typically assume steady‐state saturation and complete relaxation, leading to long scan times.

Magnetic resonance fingerprinting (MRF) is a different quantification paradigm that utilizes non‐steady state, rapidly acquired data [36]. In MRF, a simulated dictionary of synthetic signal trajectories is generated and compared (e.g., using dot‐product) to the experimentally acquired data. The best‐matching dictionary entry is then used to determine the most suitable tissue parameter set for each voxel. Several years after its introduction for water relaxometry, MRF was adapted for ST MRI [37, 38, 39, 40, 41, 42, 43]. While early ST MRF reports employed a pseudo‐random acquisition protocol [37], later studies revealed that the encoding capability is heavily dependent on the pulse sequence parameters used [43, 44]. As a result, intensive optimization is required to employ ST MRF in new applications or to shorten the scan time. Unfortunately, the extremely large size of the multi‐proton‐pool parameter space associated with ST MRI makes an exhaustive search for the optimal pulse sequence parameters impractical [43]. Several deep‐learning‐based optimization strategies have recently been developed to address this challenge [45, 46]. However, these approaches utilized the explicit closed form analytical solutions of the Bloch–McConnell equations that do not require a computationally intensive matrix exponentiation [46, 47]. Unfortunately, such expressions are not available for multi‐pool *pulsed* ST acquisition, as commonly applied in clinical scanners. This lack prevents the use of ST MRF in a variety of practical human imaging applications.

The Cramer–Rao Bound (CRB), a statistical theoretic bound for parameter estimation variance [48], has been considered previously for the optimization of quantitative water T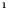 and T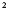 acquisition protocols [49]. Preliminary phantom studies have also indicated that it has the potential to reflect the discrimination ability of a given CEST MRF protocol [50].

Here, we describe a unified method for the automatic optimization of pulsed ST MRF acquisition protocols. Our protocol combines the CRB with a gradient‐based iterative nonlinear programming algorithm [51] and a rapid numerical BM simulator [52]. While previous implementations of the CRB in the context of T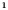/T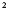 quantification used a state‐space model to derive an analytical expression for the CRB [49], we now propose a numerical approach to address the problem posed by the lack of pulsed ST analytical solutions. The method was validated using phantoms and human volunteers in a clinical 3T scanner.

## 2 Methods

### 2.1 ST MRF Acquisition Optimization Pipeline

The acquisition protocol optimization pipeline comprises three main iterative building blocks:

1. Generating a dictionary of synthetic signals for the imaging scenario of interest using a rapid BM simulator.
2. Calculating the CRB, which represents the discrimination ability and expected variance across the entire dictionary, in the context of the proton exchange parameters to be quantified.
3. Minimizing the CRB using a nonlinear programming algorithm that performs gradient‐based steps in the acquisition parameter space.

#### 2.1.1 Generation of an ST MRF Dictionary

The suggested optimization process mandates the de novo generation of a synthetic signal dictionary for any change in the acquisition parameter set. To overcome the traditionally long numerical simulation time associated with solving the Bloch–McConnell equations for a saturation pulse train, we used a Pulseq‐CEST‐based [53] C++ implemented simulator. Further acceleration and improved dictionary generation capabilities were achieved by modifying and expanding the source code using a Python interface for parallel execution [52].

The simulator implemented the numerical solution of the following equation:

(1) $$M(t+\Delta t)=(M(t)+{\text{A}}^{-1}\text{C})\cdot e^{\text{A}\Delta t}-{\text{A}}^{-1}\text{C}$$

with $T_{2}^{*}$ relaxation simulated as described in References [53, 54]. Briefly, multiple signal trajectories of subvoxel isochromats were simulated with Cauchy–Lorentz distributed $\Delta B_{0}$ inhomogeneities and summed to obtain the final magnetization signal. Additional dictionary details can be found in Supporting Information Table S1.

### 2.2 Proton Exchange Parameter Quantification

The commonly used dot‐product metric was used to reconstruct the quantitative parameter maps [37, 44]:

(2) $${\left(\hat{f_{s}},\hat{k_{sw}}\right)}_{i,j}=\underset{f_{s},k_{sw}}{\mathrm{argmax}}\frac{<e_{i,j}^{T},d(f_{s},k_{sw})>}{||e_{i,j}|{|}_{2}\cdot ||d(f_{s},k_{sw})|{|}_{2}}$$

where $e_{i,j}$ is the experimental signal trajectory at pixel (i,j) and $d(f_{s},k_{sw})$ is the dictionary entry simulated signal that corresponds to a certain proton volume fraction $f_{s}$ and exchange rate $k_{sw}$ parameter pair.

#### 2.2.1 CRB‐Guided Optimization

The main goal of the optimization process is to identify a set of acquisition parameters that enable accurate quantification of the proton exchange parameters for a given (or minimal) scan time. In the context of the CRB, this requirement translates into efficient discrimination between different signal trajectories. The optimization was performed using the sequential quadratic programming (SQP) algorithm, an iterative approach that seeks the optimum of a constrained nonlinear problem [51]. To avoid local minima (a previously observed challenge for Cramér–Rao based optimization [49]), the SQP was further combined with a basin‐hopping optimization strategy, which introduces random perturbations and “jumps” throughout the solution (acquisition parameter) space [55].

The estimation of the normalized Cramer Rao Bound [48, 50] for each synthetic signal dictionary was formulated as:

(3) $$nCRB(\theta )=\sqrt{I{\left(\theta \right)}^{-1}}/\theta$$

(4) $$\begin{array}{ll} & I(\theta )=-E\left[\frac{\partial^{2}\ln(p(x;\theta ))}{\partial \theta^{2}}\right]\overset{p≃N(s;\sigma )}{=}\\ & \frac{1}{\sigma^{2}}\overset{N-1}{\underset{n=0}{\sum }}{\frac{\partial s[n;\theta ]}{\partial \theta }}^{T}\cdot \frac{\partial s[n;\theta ]}{\partial \theta }\; \end{array}$$

where s[n] is an MRF signal trajectory simulated as the transverse part of the magnetization vector of water $s[n]=\sqrt{M_{x}^{2}+M_{y}^{2}}$ at the end of the readout. The signal differential with respect to the quantification parameters $\frac{\partial s[n;\theta ]}{\partial \theta }$ was calculated numerically as a two‐point approximation on the MRF dictionary grid. An analysis explaining the step size choice is available in Supporting Information Figures S12 and S13. The CRB was calculated with respect to $\theta =(f_{s},k_{sw})$ and constituted a 2×2 matrix. The same weighting factor was used for all tissue parameters. The optimization loss $L_{\mathrm{CRB}}$ was defined as the matrix trace:

(5) $$L_{\text{CRB}}=\text{tr}(nCRB(\theta ))$$

(6) $${\hat{\varphi }}_{\mathrm{acq}}={\text{arg min}}_{\varphi_{\mathrm{acq}}}(L_{\mathrm{CRB}})$$

where ${\hat{\varphi }}_{\mathrm{acq}}$ is the acquisition parameter matrix, which can include the saturation pulse power vector $B_{1}$[n], the frequency offset vector Δω[n], and any other acquisition parameters.

The CRB‐guided SQP pipeline was used to optimize two types of short ST MRF protocols:

i. L‐arginine CEST phantom imaging with a fixed saturation pulse frequency offset (3 ppm) and varied saturation pulse powers (0–4 μT at 3T or 0–6 μT at 7T).
ii. Semisolid MT brain imaging at 3T, with varied saturation pulse powers (0–4 μT) and frequency offsets (10–75 ppm). The data acquisition time for both protocols was restricted to less than 40 s by setting the number of raw MRF images to four or eight, while using relatively short saturation and recovery times (see Section 2.5). An initial saturation pulse pattern was randomly generated and fed into the optimization protocol, which yielded an optimized series of saturation pulse powers and frequency offsets. To assess the reproducibility and consistency of the optimization pipeline, the process was repeated at least four times for each imaging scenario and schedule length (Supporting Information Figures S1–S3) and compared across different subjects.

### 2.3 Phantom Preparation

CEST phantoms were prepared as previously described [37, 44, 56]. Briefly, L‐arginine was suspended in PBS at 25, 50, 75, 100, and 200 mM and titrated with NaOH to various pH levels between 4 and 5.5. The different solutions were placed in 2‐mL glass vials with sets of 3 vials placed into 50‐mL Falcon tubes (suitable for a preclinical scanner) or six 10‐mL vials placed inside a commercially available phantom holder (Gold Standard Phantoms, UK, model MultiSample‐120E, suitable for a clinical scanner).

### 2.4 Human Subjects

The research protocol was approved by the Tel Aviv University Institutional Ethics Board (study no. 0007572‐2) and the Chaim Sheba Medical Center Ethics Committee (0621‐23‐SMC).

Seven healthy volunteers (four males, three females, age 24.9±2.8 years) were recruited and signed an informed consent form. Four random subjects took part in the core optimization study that used randomly initialized protocols (Supporting Information Figure S3). The remaining three subjects were scanned using additional protocols, made to provide further insights related to the optimization ability following initialization with previously established reference standards [40, 45, 56], and to the effect of a super‐Lorentzian lineshape.

### 2.5 Data Acquisition

#### 2.5.1 Preclinical Continuous Wave Imaging at 7T

The L‐arginine phantoms were imaged using a preclinical 7T MRI scanner (Bruker, Germany) as a first “sanity check”, designed to ensure that the proposed optimization approach could automatically improve the parameter discrimination ability in a controlled environment, and for later comparison. A versatile 4‐, 8‐, or 30‐ raw‐images‐long MRF protocol was realized using the open‐source code described previously [52]. This comprises a continuous wave (CW) rectangular saturation pulse with saturation time (T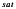) = 3 s, frequency offset Δω = 3 ppm, and recovery time (T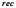) = 1 s, followed by a single‐slice SE‐EPI readout with a flip angle (FA) = 60° and echo time (TE) = 20 ms [37]. The matrix size was 64×64, and the field of view (FOV) was $32\times 32\;{\text{mm}}^{2}$.

#### 2.5.2 Clinical Pulsed Wave Imaging at 3T

The clinical scanner experiments were conducted using a 3T MRI equipped with a 64‐channel head coil (Prisma, Siemens Healthineers). All acquisition schedules were implemented using the Pulseq prototyping framework [57] and the open‐source Pulseq‐CEST sequence standard [53].

Since CW saturation is not feasible for most clinical scanners because of specific absorption rate (SAR) and physical instrumentation constraints [58], pulsed wave (PW) irradiation must be used instead. In this case, a spin‐lock saturation pulse train consisting of 13×100 ms, 50% duty cycle, with a T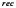 = 1 s was used [56]. The saturation block was fused with the 3D centric reordered snapshot EPI readout module described by Mueller et al. [59], providing a whole brain 1.81 mm isotropic resolution, with a FOV of $210\times 210\times 160\;{\text{mm}}^{3}$, a flip angle of 12deg, and TE = 7.8 ms.

Previously established ST MRF protocols were acquired as a reference standard method [40, 56] (Supporting Information Figure S4). These protocols used the same acquisition parameters described above, but employed a different set of saturation pulse powers and frequency offsets, and relied on a larger number of raw images (30 instead of 4 or 8).

### 2.6 Performance Evaluation and Statistical Analysis

CRB‐optimized L‐arginine concentration maps were compared to known concentrations using the mean absolute percentage error (MAPE) metric. Optimized in vitro proton exchange rate maps were compared to steady‐state quantification of the exchange rate QUESP‐derived values [32], as described previously [37].

In the absence of absolute ground truth in vivo, the CRB‐optimized proton volume fraction and exchange rate maps were compared to maps obtained using a reference standard MRF protocol [40, 52]. Quantification performance in vivo was estimated using the normalized root mean squared error (NRMSE) metric:

(7) $$\text{NRMSE}(I_{\text{GS}},I_{S})=\frac{\text{RMSE}(I_{GS},I_{S})}{\max(I_{GS})-\min(I_{GS})},$$

where $I_{GS}$ is the reference map and $I_{S}$ is a map quantified using a proposed (CRB‐optimized) sequence. We also calculated the structural similarity index metric (SSIM) and the Pearson correlation coefficient.

Statistical analysis used a two‐tailed paired t‐test, implemented with the open‐source SciPy scientific computing library for Python [60] and presented as box plots. Statistics in the text are presented as mean ± SD. Differences were considered significant at p<0.05. Asterisk notations were designated as *p<0.05, **p<0.01, and ***p<0.001.

## 3 Results

### 3.1 CW Phantom Imaging at 7T

**FIGURE 1:**
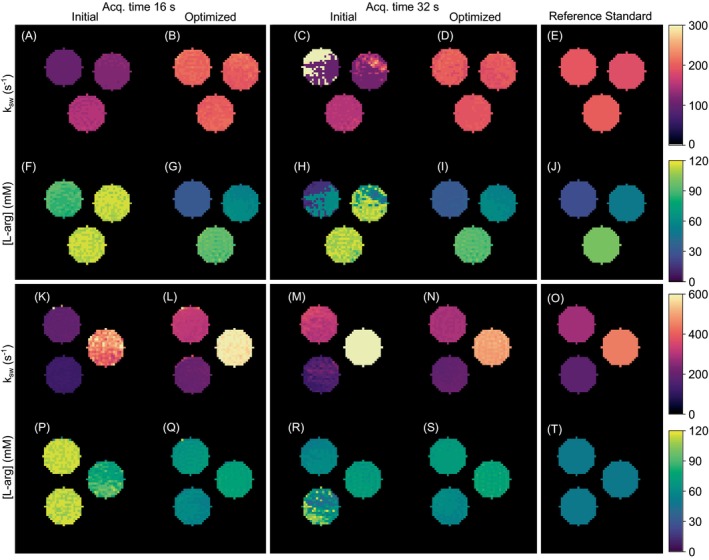
CW phantom imaging at 7T. (**Left**): Representative two pairs of pre (**A, F, K, P, C, H, M, R**) and post (**B, G, L, Q, D, I, N, S**) CRB optimization L‐arginine concentration and proton exchange maps, for an MRF schedule that acquires 4 or 8 raw images (in 16 s and 32 s, respectively). (**Right**): Ground truth concentrations (**J, T**) and QUESP‐derived (**E, O**) reference exchange rates [37].

**FIGURE 2:**
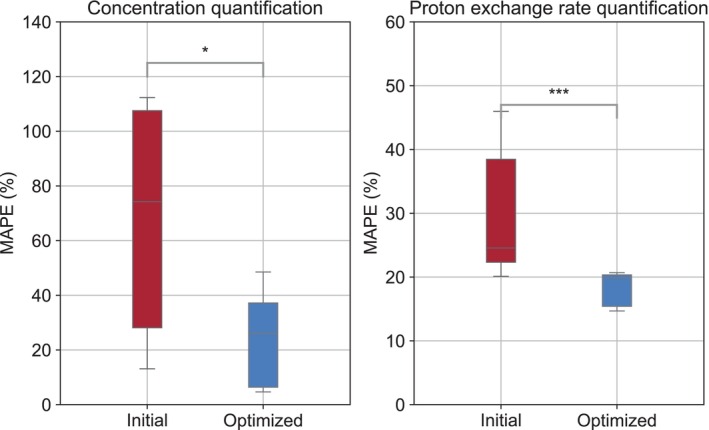
Statistical analysis of the mean absolute percent error (MAPE) across different L‐arginine vials imaged at 7T. *p<0.05; ***p<0.001.

Two representative comparisons of the L‐arginine concentration and proton exchange rate maps obtained using the randomly generated and CRB‐optimized acquisition protocols at 7T are shown in Figure 1. Additional preclinical parameter maps obtained following the CRB optimization with six different random sequence initializations are available in Supplementary Information Figure S5. After optimization, MAPE dropped from 85.3%±71.2% to 23.2%±16.5% for L‐arginine concentration mapping (p<0.05) and from 30.4%±9.3% to 17.9%±5.5% for proton exchange rate mapping (p<0.001, Figure 2). The acquisition times for the optimized protocols were 16 s and 32 s for acquiring four or eight raw MRF images, respectively.

### 3.2 PW Phantom Imaging Using a Clinical 3T Scanner

**FIGURE 3:**
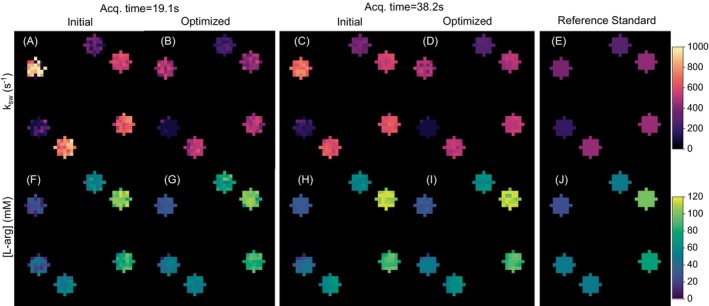
PW phantom imaging at 3T. (**Left**): A representative pair of pre (**A, F, C, H**) and post (**B, G, D, I**) CRB‐optimization L‐arginine proton exchange rate (**top**) and concentration (**bottom**) maps for an MRF schedule that acquires 4 or 8 raw images (in 19.1 s or 38.2 s, respectively). (**Right**): QUESP‐derived reference exchange rates [37] (**E**) and ground truth concentrations maps (**J**).

**FIGURE 4:**
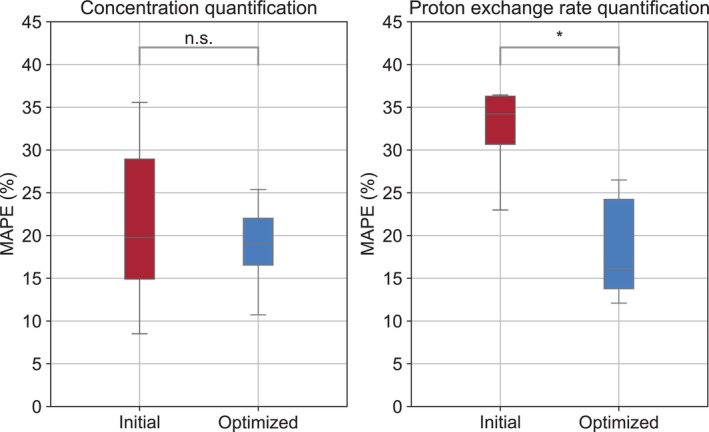
Statistical analysis of the mean absolute percent error (MAPE) across different L‐arginine vials imaged using a PW MRF pulse sequence at a 3T clinical scanner. *p<0.05.

A representative comparison of L‐arginine concentration and proton exchange rate maps obtained using randomly generated and CRB‐optimized PW acquisition protocols at 3T is shown in Figure 3. The parameter maps obtained from all six optimization attempts with different random sequence initialization are available in Supporting Information Figure S6. The MAPE for the proton exchange rate estimation decreased from 34.8%±8.5% to 21.4%±11.1% (p<0.05, Figure 4). Although the MAPE for estimating the L‐arginine concentration decreased from 21.5%±9.5% to 18.8%±4.7%, the effect was not significant (p = 0.34). The acquisition time was 19.1 s and 38.2 s for the MRF protocols that acquired four and eight raw images, respectively.

### 3.3 PW Human Brain Imaging at 3T

**FIGURE 5:**
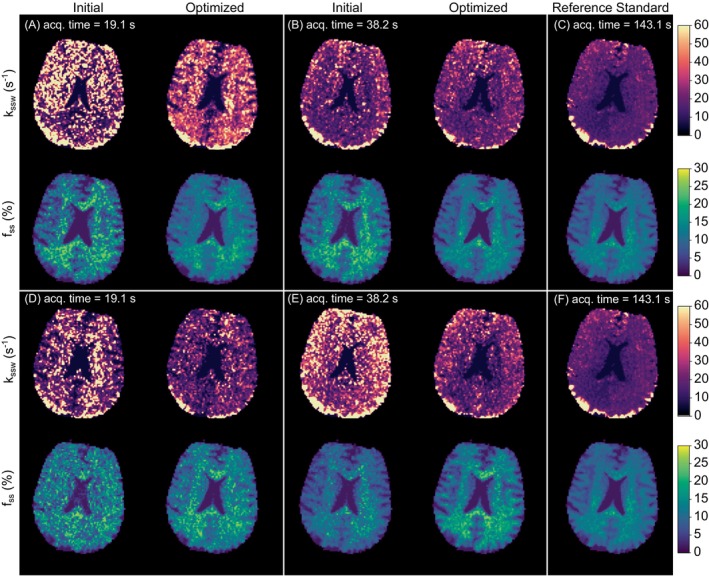
PW ST MRF imaging in a healthy human volunteer at 3T. **(A, B, D, E)** Pre‐ and post‐CRB‐optimization parameter maps from four independent procedures, each initiated using a randomly generated acquisition protocol (Supplementary Information Figure S3). **(C, F)** Reference maps obtained using a previously established (and longer) acquisition protocol [40, 56].

**FIGURE 6:**
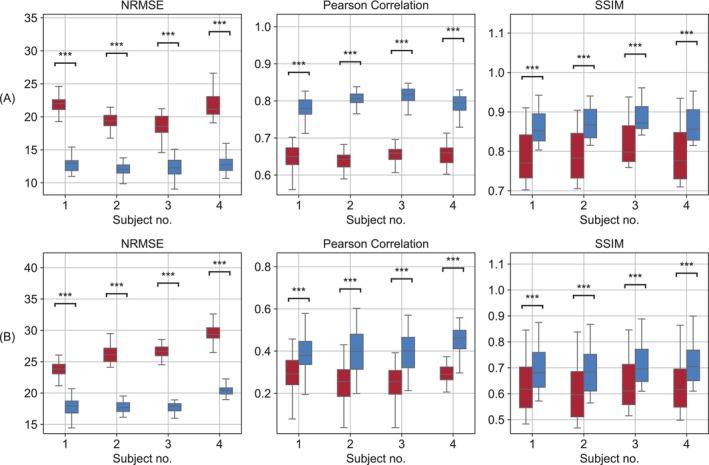
Statistical analysis of the in vivo semisolid MT proton volume fraction (**A**) and proton exchange rate (**B**) quantification performance. The NRMSE, SSIM, and Pearson's correlation values were calculated with respect to reference maps obtained using a previously established (and longer) acquisition protocol [40, 56]. The red and blue box plots represent the initial and CRB‐optimized acquisition protocols, respectively.

Four pairs of pre‐ and post‐CRB optimization parameter maps from a representative subject are shown in Figure 5. All the in vivo parameter maps obtained using twelve different random initializations are available in Supporting Information Figures S7–S10. In general, the optimization pipeline provided an improved signal‐to‐noise ratio (SNR) and produced images that were less noisy and more visually similar to the reference ground truth maps obtained using a previously established (and longer) acquisition protocol [40, 56] (Figure 5C,F). A statistical analysis based on all optimization attempts, subjects, and slice images is provided in Figure 6. The CRB‐guided semisolid MT volume fraction (f_*ss*_) maps demonstrated better correlation with the reference standard than the maps obtained using randomly generated protocols (Pearson's r = 0.79 ± 0.03 compared to 0.64 ± 0.04), a higher SSIM (0.87 ± 0.04 compared to 0.80 ± 0.06), and a lower NRMSE (12%±1% compared to 20%±2%). Similarly, the proton exchange rate (k_*ssw*_) maps obtained using the CRB‐optimized protocols demonstrated higher SSIM (0.70 ± 0.08 compared to 0.63±0.10), increased Pearson's correlation (0.41±0.09 compared to 0.26±0.09), and a lower NRMSE (20%±5% compared to 28%±8%). Importantly, all the improvements in metric performance described above (for both f_*ss*_ and k_*ssw*_) were statistically significant for all subjects (p<0.001, Figure 6). The acquisition time was identical to the PW phantom case, namely 19.1 s and 38.2 s for the MRF protocols that acquired four and eight raw images, respectively.

Four pairs of pre‐ and post‐CRB optimization parameter maps from a representative subject are shown in Figure 5. All the in vivo parameter maps obtained using twelve different random initializations are available in Supporting Information Figures S7–S10. In general, the optimization pipeline provided an improved signal‐to‐noise ratio (SNR) and produced images that were less noisy and more visually similar to the reference ground truth maps obtained using a previously established (and longer) acquisition protocol [40, 56] (Figure 5C,F). A statistical analysis based on all optimization attempts, subjects, and slice images is provided in Figure 6. The CRB‐guided semisolid MT volume fraction (f_*ss*_) maps demonstrated better correlation with the reference standard than the maps obtained using randomly generated protocols (Pearson's r = 0.79 ± 0.03 compared to 0.64 ± 0.04), a higher SSIM (0.87 ± 0.04 compared to 0.80 ± 0.06), and a lower NRMSE (12% ± 1% compared to 20% ± 2%). Similarly, the proton exchange rate (k_*ssw*_) maps obtained using the CRB‐optimized protocols demonstrated higher SSIM (0.70 ± 0.08 compared to 0.63 ± 0.10), increased Pearson's correlation (0.41 ± 0.09 compared to 0.26 ± 0.09), and a lower NRMSE (20% ± 5% compared to 28% ± 8%). Importantly, all the improvements in metric performance described above (for both f_*ss*_ and k_*ssw*_) were statistically significant for all subjects (*p* < 0.001, Figure 6). The acquisition time was identical to the PW phantom case, namely 19.1 s and 38.2 s for the MRF protocols that acquired four and eight raw images, respectively.

## 4 Discussion

The methodological approach described here is designed to enable PW ST MRF optimization by harnessing the CRB to guide a nonlinear iterative optimization that leverages a rapid numerical BM simulator.

Notably, while most clinical CEST studies to date utilized PW saturation [24], several CEST‐weighted [61, 62], and even ST MRF studies [63] have implemented a pseudo‐CW 100% duty cycle saturation using parallel RF transmission (pTx). However, implementing advanced pTx often requires a careful vendor‐specific sequence programming [64, 65].

An initial sanity check with CW‐scanned phantoms revealed that applying the optimization pipeline consistently improved quantification accuracy (Figures 1, 2, and Supporting Information Figure S5). Similarly, a phantom experiment under PW conditions in a clinical 3T scanner provided better encoding capabilities following optimization (Figure 3). Notably, most of the improvement achieved was a result of more accurate quantification of the proton exchange rate (Figure 4), since the L‐arginine concentration maps for the randomly generated protocols were already in reasonable agreement with the ground truth (Figure 3, bottom panel).

In vivo, there was a marked improvement in visual similarity between the reconstructed quantitative parameter maps and the reference standard method, following CRB‐based optimization (Figures 5, 6). The improvement was most visually discernible for the very short acquisition protocols (scan time = 19.1 s, Figure 5A,D), where the parameter maps obtained for the randomly generated protocols were very noisy.

To gain a basic intuition of the decisions made by the optimizer, we performed a meta‐analysis comparing the distribution of the saturation pulse parameters used by the randomly initialized and the CRB‐optimized acquisition schedules (Supplementary Information Figure S11). The standard deviation of the optimized saturation pulse powers was significantly increased compared to the baseline in all cases (p<0.05). This can be reasoned by an improved encoding capability associated with using a wider range of powers, facilitating a more efficient saturation in various proton exchange rates. Some of the optimized acquisition patterns have further tended to favor values at the upper or lower bound of the parameter range, as can be more easily seen when the optimizer is forced to produce a very short schedule (e.g., composed of only four raw images, Supporting Information Figure S1, left). This behavior is in line with previous MRF optimization efforts [49, 66]. The variance of the saturation pulse frequency offset was not significantly higher following optimization, which can be explained by the broad spectral linewidth associated with the semisolid MT proton, which creates various “opportunities” for sufficient encoding, regardless of spanning a vast frequency offset range. The change in the mean saturation pulse parameters following optimization did not present a clear and consistent trend across all imaging cases. The changes in mean saturation parameter value were only significant for the CW saturation pulse power optimization (p<0.001).

The main challenges in interpreting PW MRF in clinical scanners compared to CW MRF in preclinical scanners are the reduced saturation efficiency (due to the lower duty cycle), the more difficult modeling of the saturation pulse train, and the larger field inhomogeneities. Although the field heterogeneity issue was mitigated here by simulating multiple subvoxel isochromats with Cauchy–Lorentz distributed $\Delta B_{0}$ inhomogeneities, future work could explicitly input experimentally measured field inhomogeneities in the per‐voxel quantification [40].

Previous studies have shown that saturation‐encoded sequences may be sensitive to B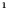 inhomogeneity [67, 68, 69]. One of the limitations of our study is that this effect was not accounted for during the optimization pipeline. To nonetheless analyze the impact of B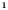 inhomogeneity, we performed a separate quantification attempt, where the proton exchange maps were corrected by accounting for the ground truth (WASABI [70] protocol‐driven) B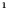 values in a pixelwise manner. While the results demonstrated a consistent improvement in encoding capability compared to the initialized random acquisition protocol (Supporting Information Figure S14), the performance metrics were not substantially different than those obtained without B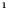 correction (Figure 6). Although incorporating B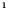 variability during the optimization process would considerably increase the computation time, it is expected to be further imperative at higher field strengths or when using surface‐coil excitation [67].

In Figure 5 and Supporting Information Figures S7–S10, relatively high apparent exchange rates were observed at the occipital cortex. As this effect was present across all subjects, it is likely related to the site hardware (coil‐related artifact) and could be corrected using previously reported techniques [71].

This study deliberately used a very short (less than 40 s) pulse sequence, which generates only four or eight raw MRF images. The rationale was to explore the limits of ST MRF acceleration and potentially discover an optimized acquisition schedule for rapid and quantitative preliminary screening of ST effects using clinical scanners. While the post‐optimization performance metrics were adequate for semisolid MT volume fraction mapping (Figure 6, top), the proton exchange rate quantification yielded a lower correlation with the reference maps obtained using a previously established and longer MRF protocol (30 raw images). This can be attributed to the well‐known challenge of accurately quantifying the noisy semisolid MT proton exchange rate, where relatively subtle changes are spatially manifested across the brain [43, 72].

The consistency of the optimization results was evaluated by calculating the coefficients of variance (CoV) of the estimated parameters in the WM/GM ROIs of human subjects (Supporting Information Table S3). The assessment was performed on twelve different random protocol initializations (Supporting Information Figure S3), five full‐length and shortened previously established MRF protocols [40, 45, 56] (Supporting Information Figures S21 and S23), and their CRB‐optimized counterparts. In the majority of cases, the CoV was lower (improved) following CRB optimization. As expected, the CoV was lower for longer acquisition protocols (e.g., CoV for f_*ss*_ = 4.59%–5.72% for an acquisition time of 38.2 s compared to CoV for f_*ss*_ = 8.54%–17.88% for an acquisition time of 19.1 s). The worst CoV values were obtained for the semisolid MT proton exchange rate (k_*ssw*_) estimation, specifically when very short acquisition protocols (19.1 s) were used. This can be explained by the substantial noise manifested in the proton exchange rate maps acquired using drastically short protocols (Figure 5, left).

To further explore the noise robustness of the optimized schedules, we repeated the quantification process following an independent denoising of the raw MRF data using Marchenko–Pastur principal component analysis (MPPCA) [73]. Interestingly, while only relatively small differences were apparent in the raw images and trajectories (Supporting Information Figure S15a–d,i–l), noticeable changes were obtained in the output maps (Supporting Information Figure S15e–h,m–p) which led to an improvement in the quantification performance of both the randomly initialized and the CRB‐optimized protocols (Supporting Information Figure S16, especially for the proton exchange rate maps). Therefore, at least a portion of the quantification improvement obtained by the optimization procedure can be associated with improved noise robustness. The remaining difference (Supporting Information Figure S16) can be attributed to the improved encoding capability of the CRB‐optimized acquisition schedules.

The super‐Lorentzian lineshape is considered a better approximation for the semisolid MT proton pool compared to the Lorentzian lineshape [74, 75, 76]. Our decision to use the latter was driven by its ability to be simulated faster. To analyze the influence of the lineshape on the quantification accuracy, we have generated new signal dictionaries with a super‐Lorentzian lineshape. The dictionaries were then matched to raw data derived from both a representative optimized (and short) acquisition protocol, and to the raw images taken using the reference (and long) acquisition protocol [40, 56]. In the CRB‐optimized and short schedule, replacing the Lorentzian with super‐Lorentzian has led to subtle differences in the proton volume fraction map (Supporting Information Figure S17a,c), but improved the proton exchange rate WM/GM contrast (Supporting Information Figure S17b,d). For the full‐length reference protocol, relatively minor changes were observed when the lineshape was modified (Supporting Information Figure S17e–h). To further demonstrate that the same optimization approach is amenable to the prospective use of various lineshapes, we performed another optimization study from scratch, where a short random protocol of four raw images was initialized, and optimized using the suggested CRB method and a super‐Lorentzian lineshape. The resulting quantitative maps (Supporting Information Figure S18) demonstrate that the CRB‐optimization introduces an improvement in SNR, WM/GM contrast, and resemblance to reference data compared to the unoptimized sequences, supporting the future incorporation of the super‐Lorentzian lineshape for improved quantification accuracy.

Existing ST MRF acquisition protocols, as described in several previous works [40, 56] have already demonstrated utility for APT and semisolid MT imaging. These protocols were previously validated across phantoms, mice, and humans, and were further used in several follow‐up publications [43, 52, 77]. The main goal of this work was not to improve upon their accuracy, but rather to provide a numerically viable computational approach that will address the need for a *PW‐compatible*, ST MRF optimization technique. Such optimization enables the use of shorter acquisition schedules and could also be potentially amenable to other future efforts, including different target organs, other CEST compounds, and different imaging scenarios.

When using the full length previously established reference standard protocol [40, 56] as the initialization for the CRB optimization method, the parameter maps obtained are highly similar to those of the reference (Supporting Information Figure S19e,f,k,l, Supporting Information Figure S21l,p,m,q), as expected. However, a serial shortening of the reference standard, when serving as an initialization for the CRB method, demonstrates the importance of the optimization for accelerated imaging (Supporting Information Figures S19–S24).

The numerical nature of the proposed approach makes it suitable for a variety of pulse shapes and any number of proton pools, with the primary cost being the optimization time. The CRB‐SQP process took between 5 to 31 h on a single desktop, depending on the imaging scenario (Supplementary Table S2). In this context, recent studies have shown that multi‐pool CEST imaging necessitates the serial acquisition of T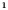, T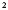, semisolid MT and CEST encoded data and subsequent integration in a quantification scheme that combines the entire information [40, 42]. Although we focused here on in‐vivo semisolid MT quantification and a proof‐of‐concept in‐vitro CEST imaging, future research will be needed to validate the use of the CRB‐based optimization in more complex imaging scenarios (and pathologies).

An important factor in the success of the in‐silico pre‐scan offline optimization was the rapid BM simulator used, which not only incorporated C++ and Python backend parallelization for computational efficiency [52], but was also fully compatible with Pulseq‐CEST standards. This enabled an accurate realization of the same PW saturation properties, as played out at the scanner [53].

This proof of concept study optimized two main acquisition parameters (Supporting Information Figures S1–S3): The saturation pulse power ($B_{1}$) and the frequency offset (Δω). However, the proposed pipeline can be readily extended to optimize additional parameters, such as the saturation pulse length, flip angle, and recovery time.

Another possibility for improvement is that here, we employed the classical dot product between the experimentally measured trajectories and the simulated dictionary entries as the quantification metric [36]. While the dot‐product is often used and is simple to implement, it is possible that alternative NN‐based quantification approaches [40, 41, 72, 78] could provide improved accuracy. Interestingly, a recent study has shown that the CRB can be further utilized to improve such NN reconstruction by participating in the loss function normalization [79].

## 5 Conclusion

The CRB‐based optimization framework demonstrates the ability to accelerate 3D acquisitions of semisolid MT and CEST mapping by 3.75 to 7.5 fold, with a better performance than pseudo‐randomly generated protocols. The unlocked optimization ability for pulsed saturation creates new opportunities to support future clinical ST research.

## Conflicts of Interest

The authors declare no conflicts of interest.

## Supporting information




**Data S1**: Supporting Information.

## Data Availability

The source code, phantom data, and 2D human sample data are publicly available at https://github.com/momentum‐laboratory/crb‐optim. The reference L‐arginine and semisolid MT MRF pulse sequences are available at the Pulseq online library [53, 80]. The preclinical and all CRB‐optimized pulse sequences can be reproduced using the open‐source software described previously [52] and the parameters defined in Supplementary Information Figures S1–S3.
