# Supplementary material for: Optimization of Pulsed Saturation Transfer MR Fingerprinting (ST MRF) Acquisition Using the Cramér–Rao Bound and Sequential Quadratic Programming
Source: Magn Reson Med. 2025 Oct 18;95(3):1778–90. doi: 10.1002/mrm.70141 (PMC12746367; doi:10.1002/mrm.70141)
Supplement: Supplementary file 1 — Data S1: Supporting Information. [file MRM-95-1778-s001.pdf]

Supporting Information

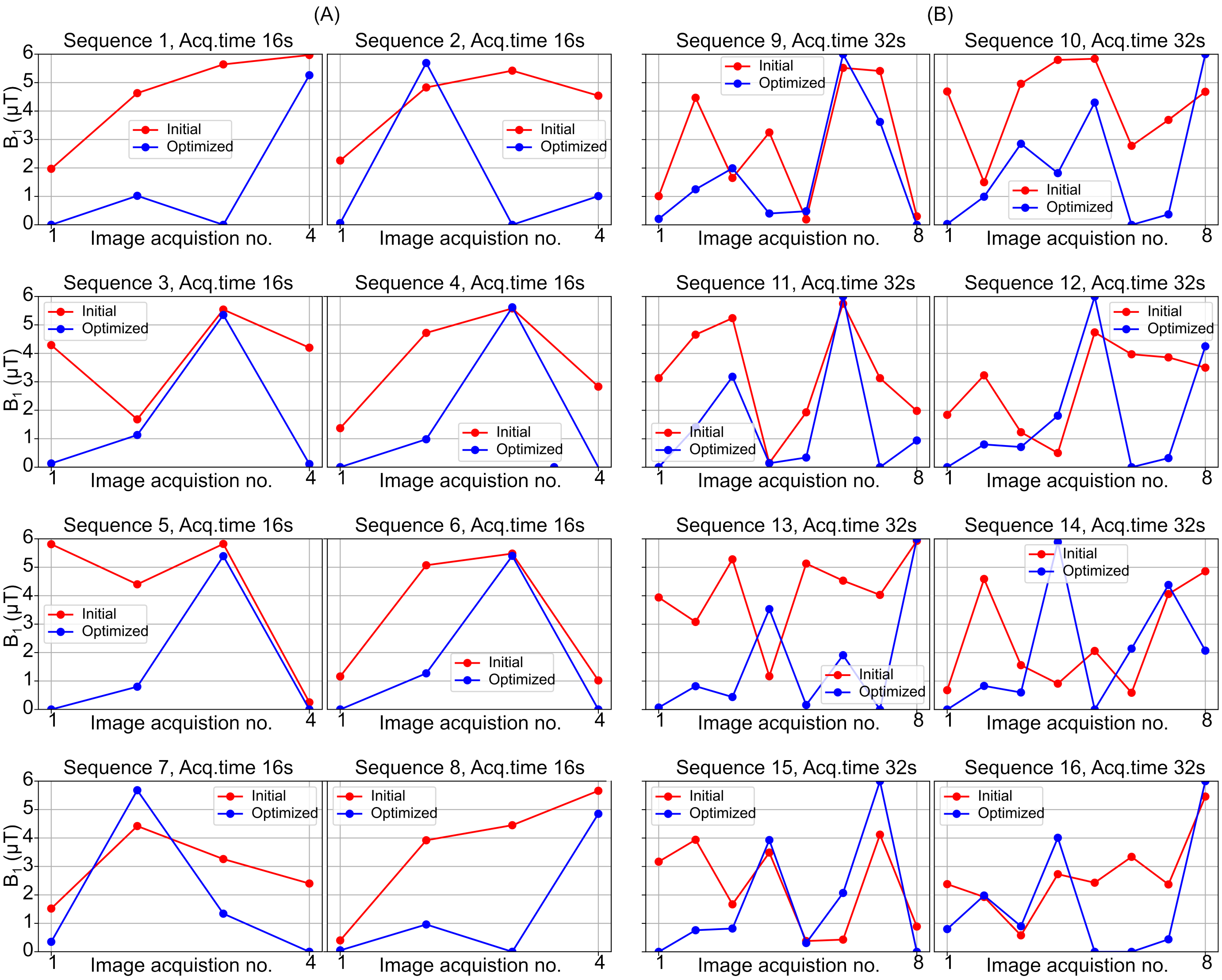

Supporting Information Figure S1. Initial and optimized acquisition schedules for CW L-arginine phantom imaging at a preclinical 7T scanner. (**A**) Schedules aimed to acquire four raw MRF images (acquisition time = 16 seconds). (**B**) Schedules aimed to acquire eight raw MRF images (acquisition time = 32 seconds).

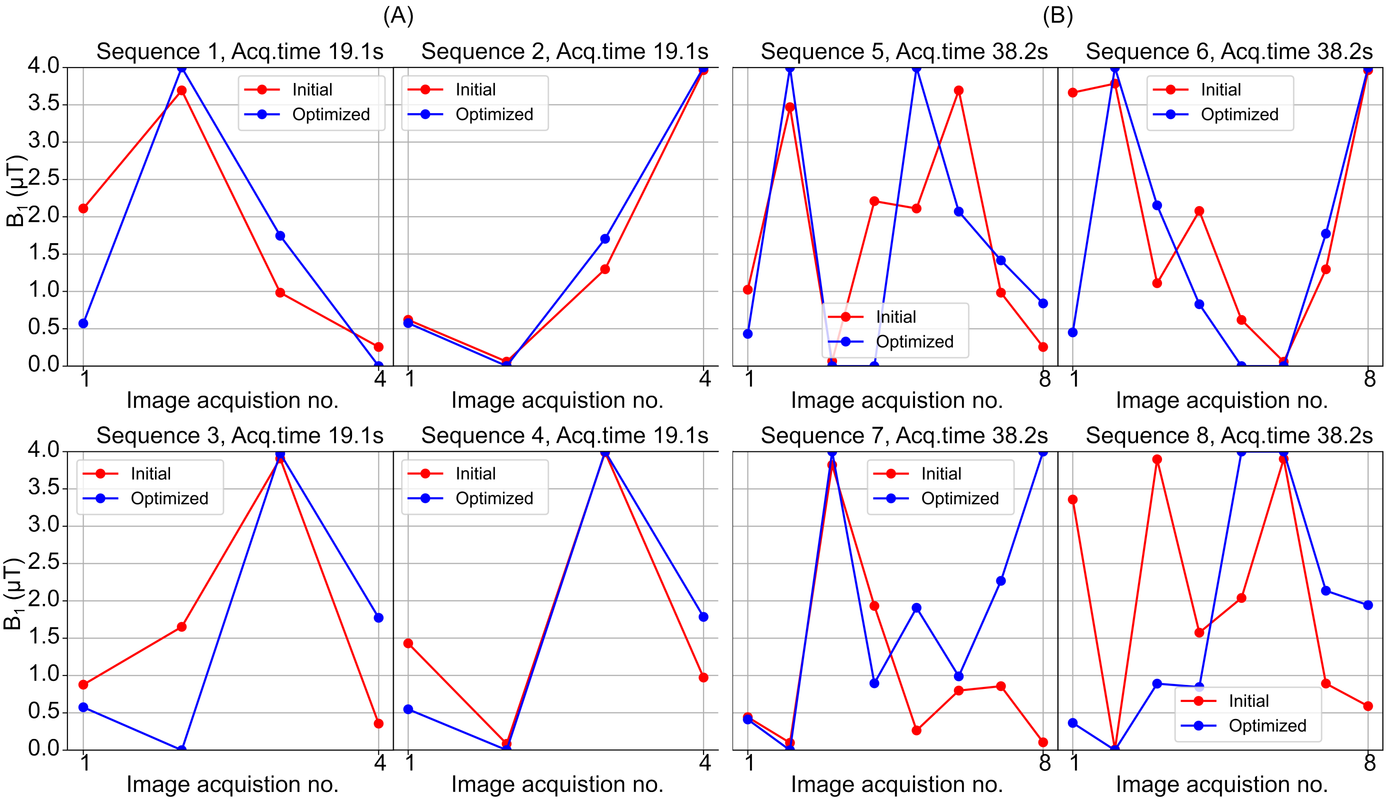

Supporting Information Figure S2. Initial and optimized acquisition schedules for PW L-arginine phantom imaging at a clinical 3T scanner. (**A**) Schedules aimed to acquire four raw MRF images (acquisition time = 19.1 seconds). (**B**) Schedules aimed to acquire eight raw MRF images (acquisition time = 38.2 seconds).

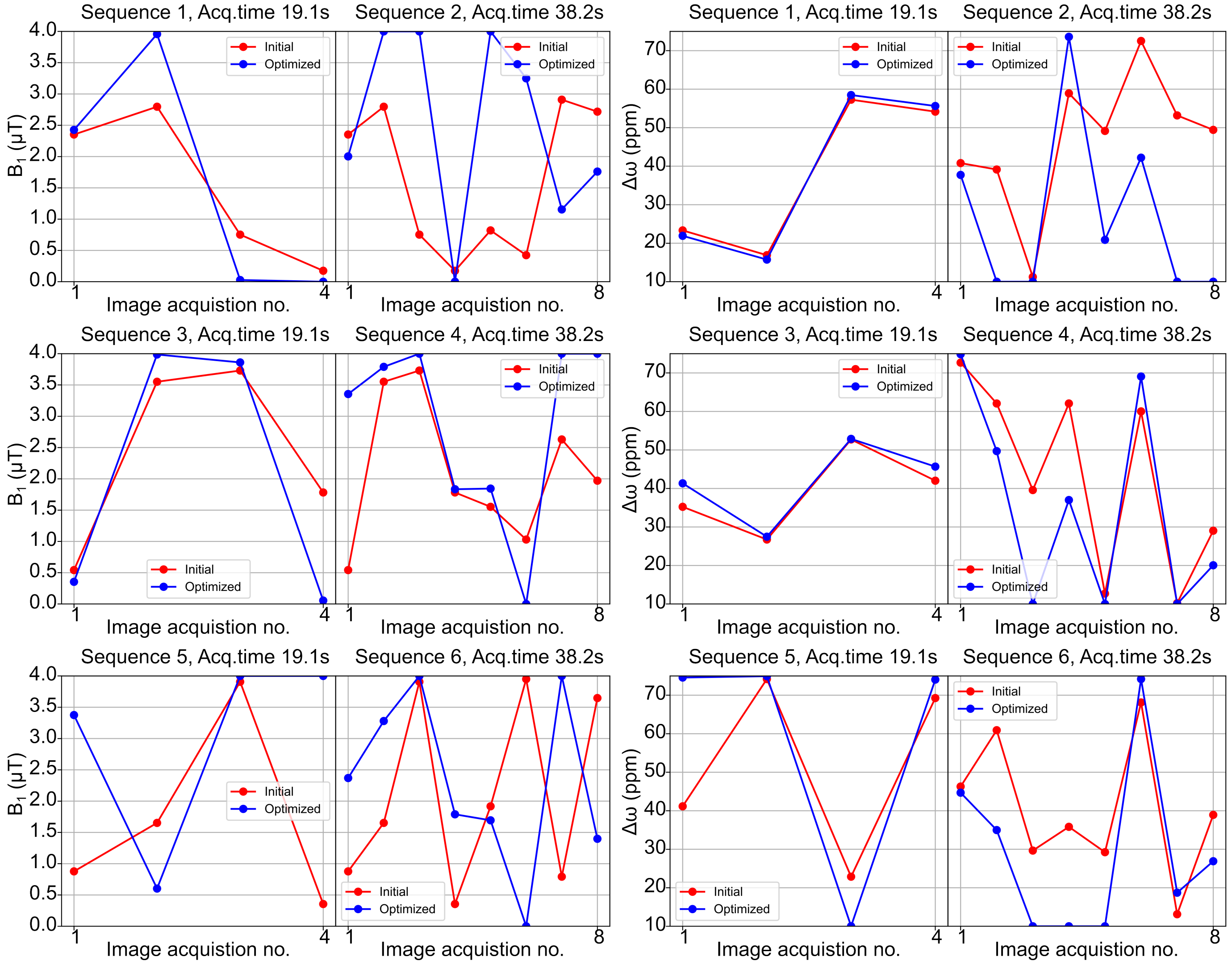

Supporting Information Figure S3. Initial and optimized acquisition schedules for PW in vivo human imaging at a 3T scanner, where both the saturation pulse power (B_1, left) and frequncy offset (Δw, right) were optimized.

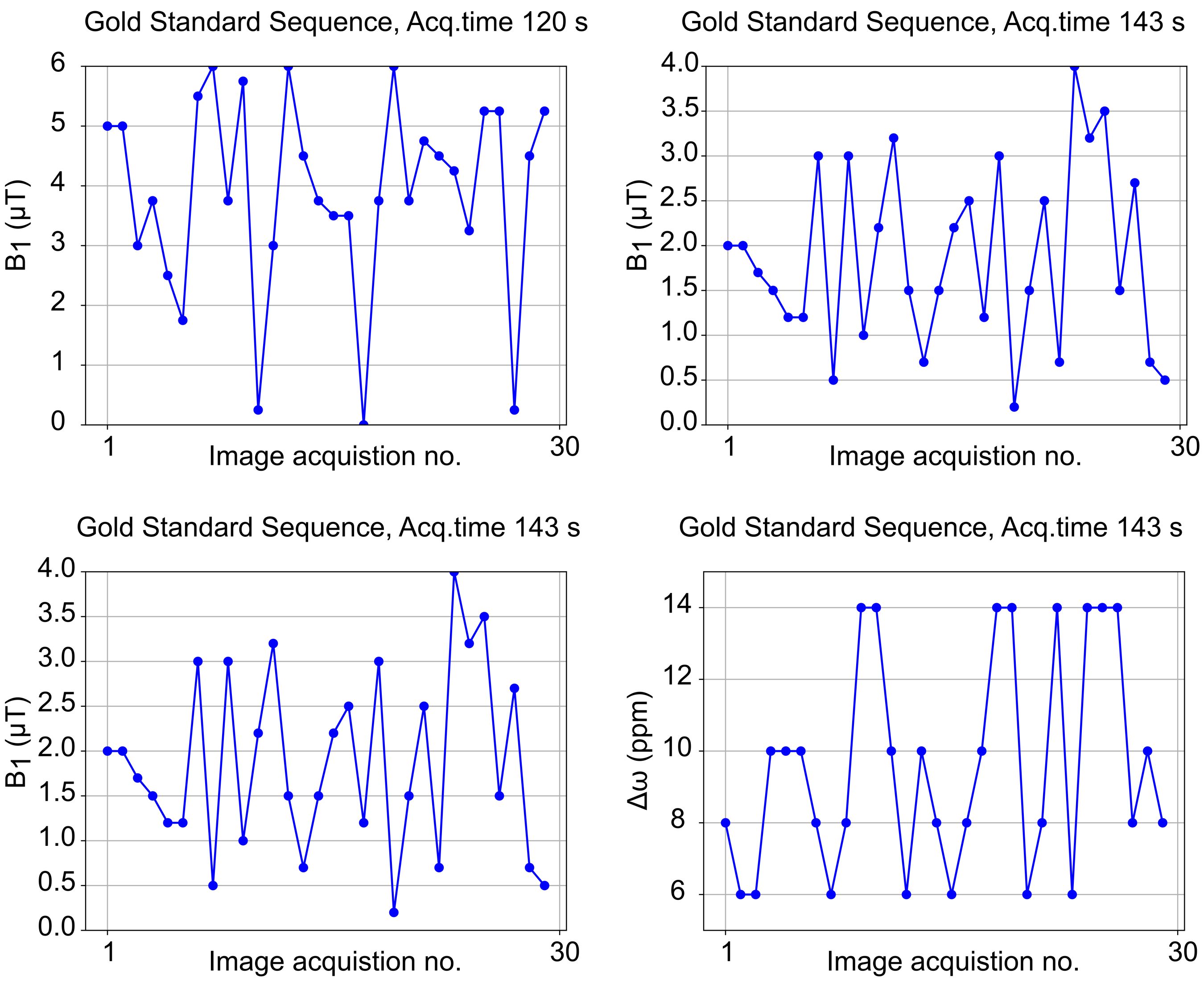

Supporting Information Figure S4. Reference standard MRF acquisition protocols. (Top left). The saturation pulse powers used for CW phantom imaging in previously established reports[37]. A total of 30 images were acquired with a total acquisition time = 120 s. **(Top right)**. The saturation pulse powers used for PW phantom imaging[40]. A total of 30 images were acquired with a total acquisition time = 143 s). **(Bottom left)**. The saturation pulse powers and (**Bottom right**) frequency offsets used for PW human imaging[40]. A total of 30 images were acquired with a total acquisition time = 143 s. All other acquisition parameters are described in section 2.4.

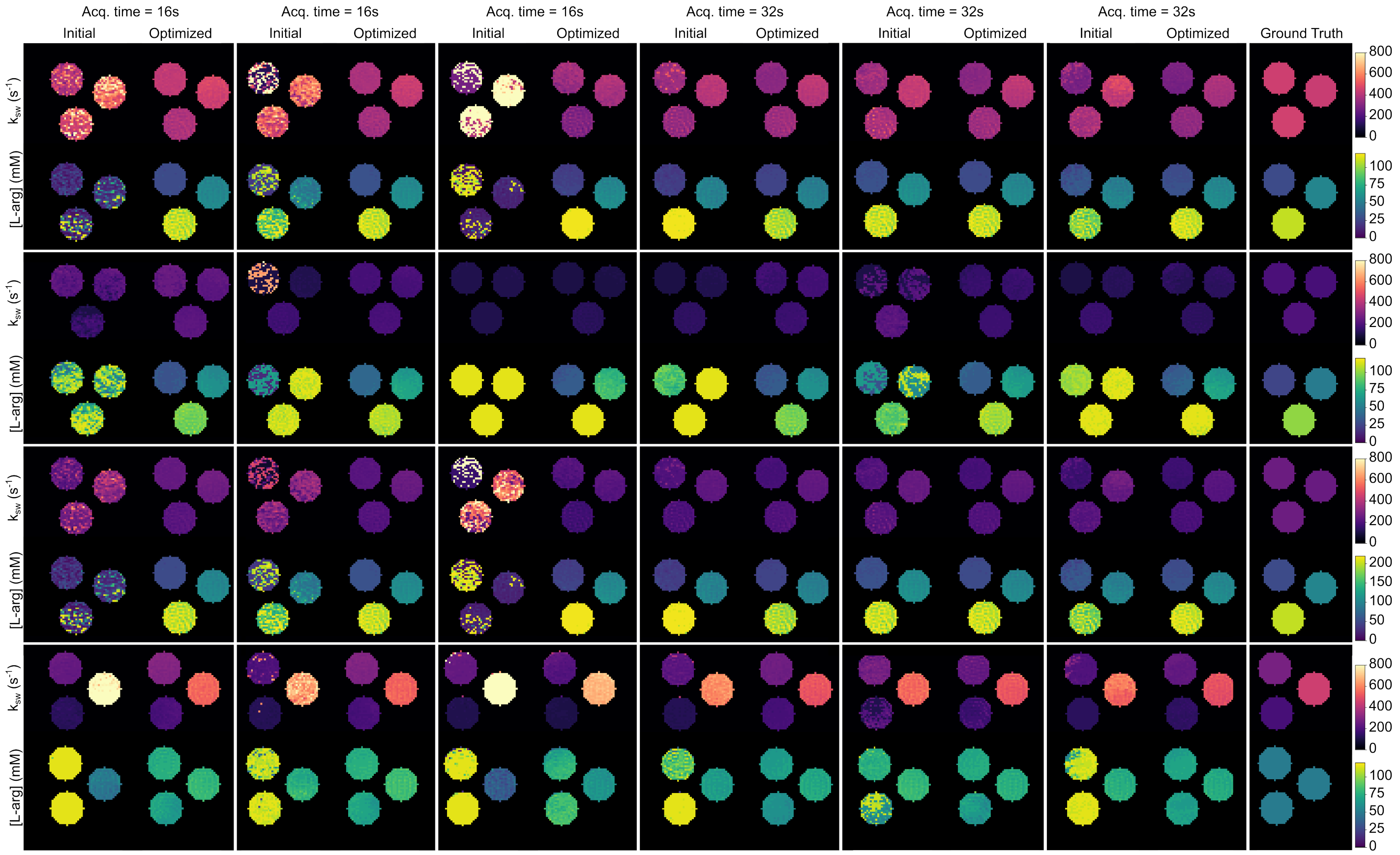

Supporting Information Figure S5. Six additional examples for CW phantom imaging at 7T, before and following CRB-based optimization. Each column contains the quantification maps obtained by applying the same initial and optimized pulse sequence pair across four different phantoms.

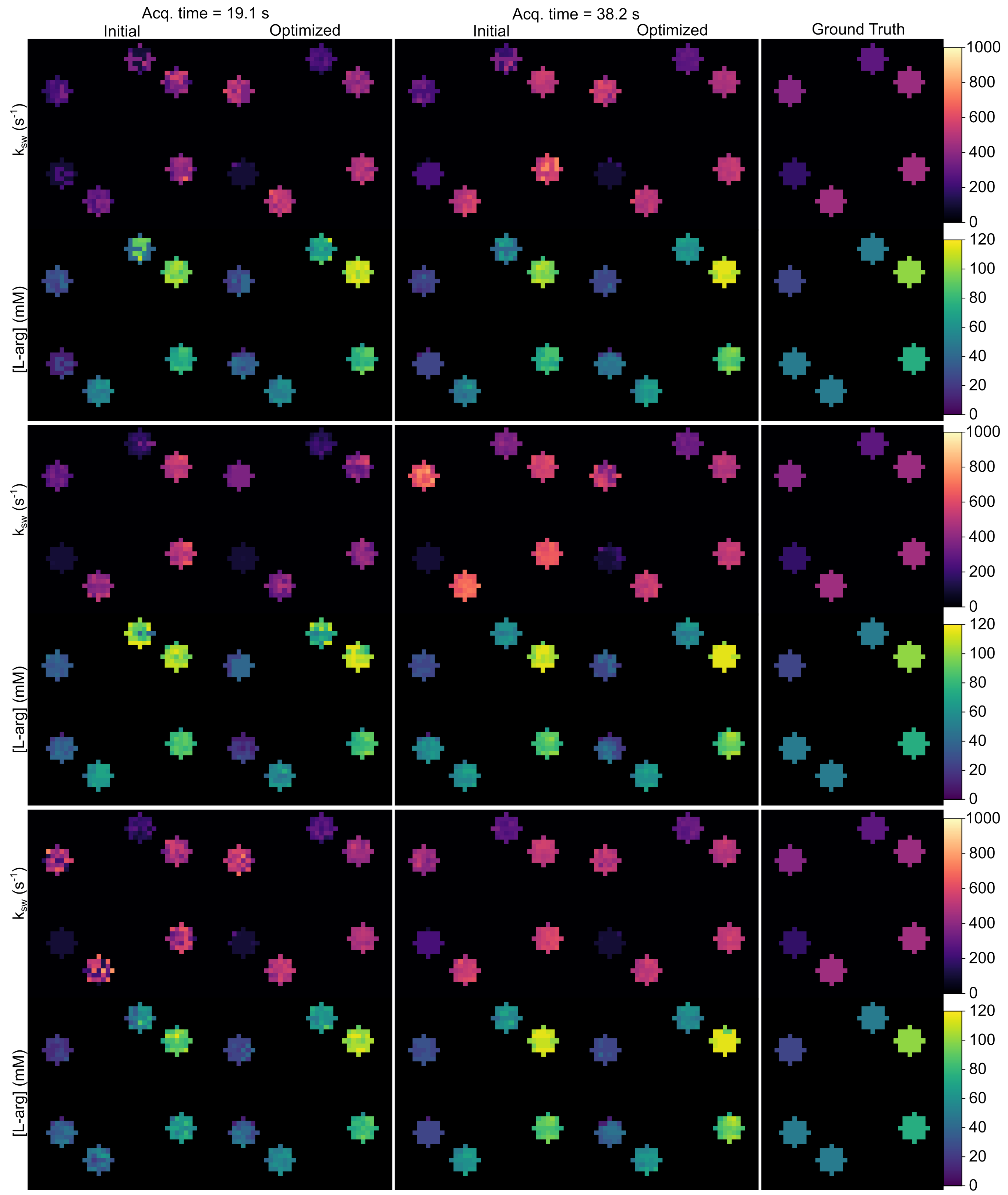

Supporting Information Figure S6. The initial and optimized output parameter maps following all six optimization procedures performed for PW phantom imaging at 3T.

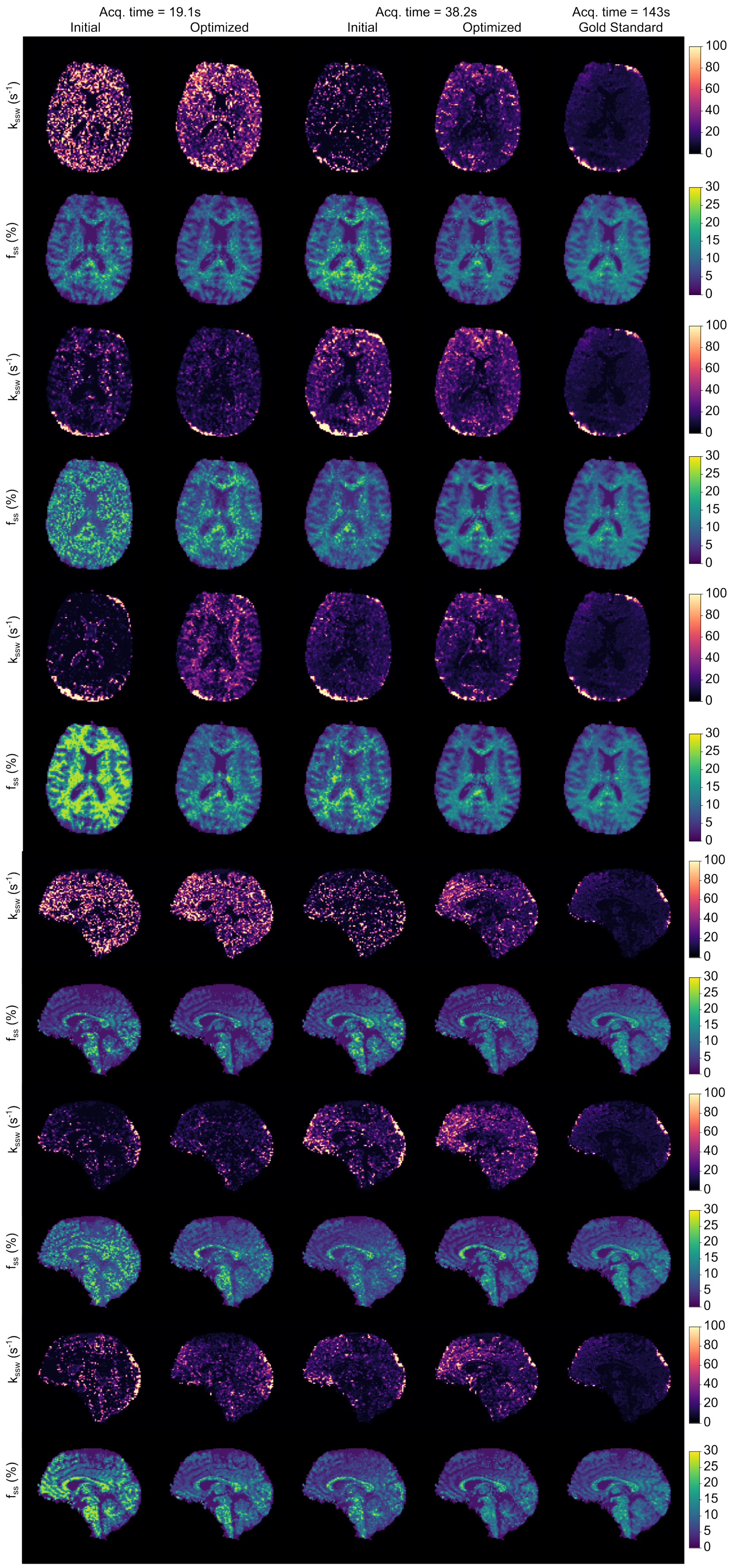

Supporting Information Figure S7. PW ST MRF imaging in healthy human volunteer #1. A representative slice is shown for each of the twelve optimization procedures performed (see Figure S3).

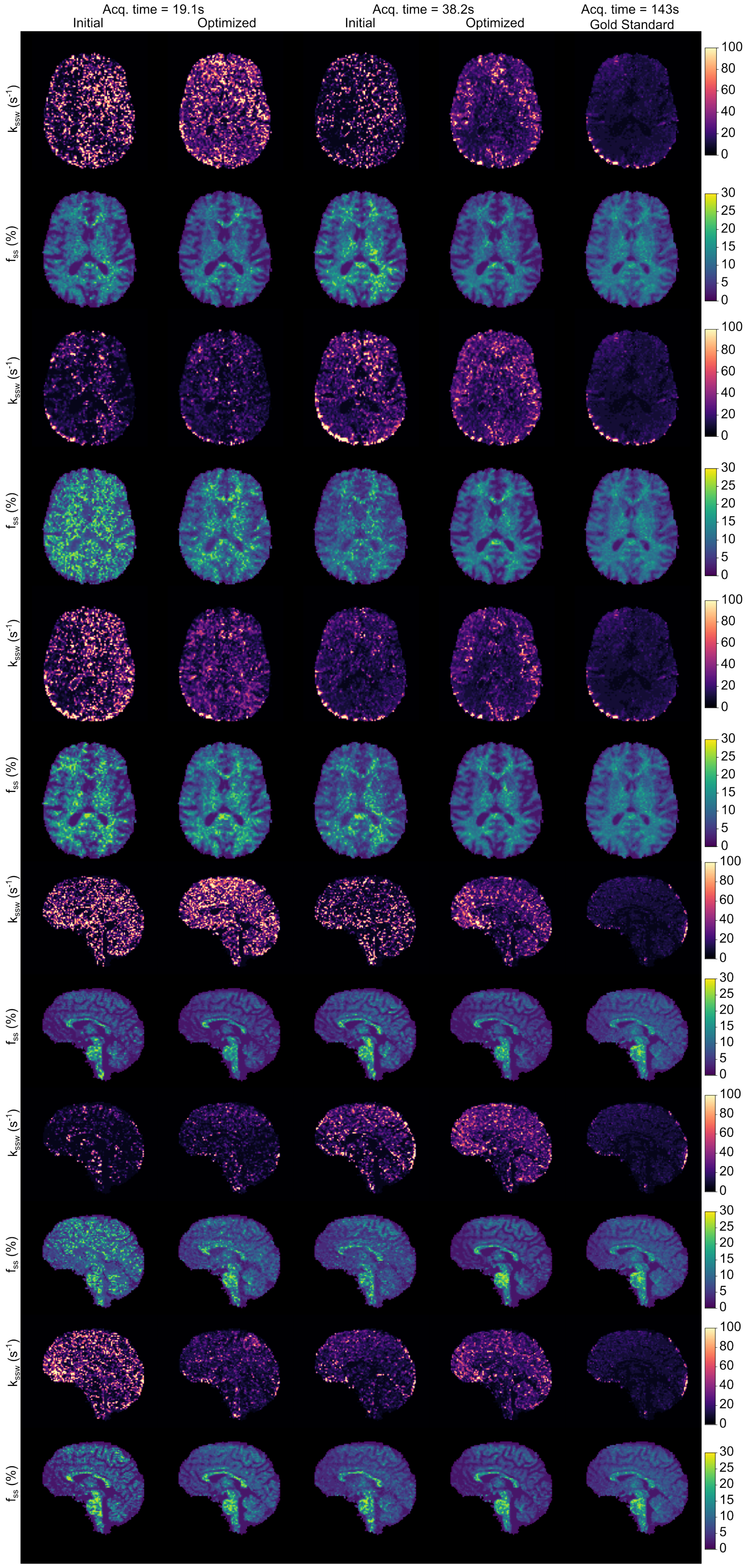

Supporting Information Figure S8. PW ST MRF imaging in healthy human volunteer #2. A representative slice is shown for each of the twelve optimization procedures performed (see Figure S3).

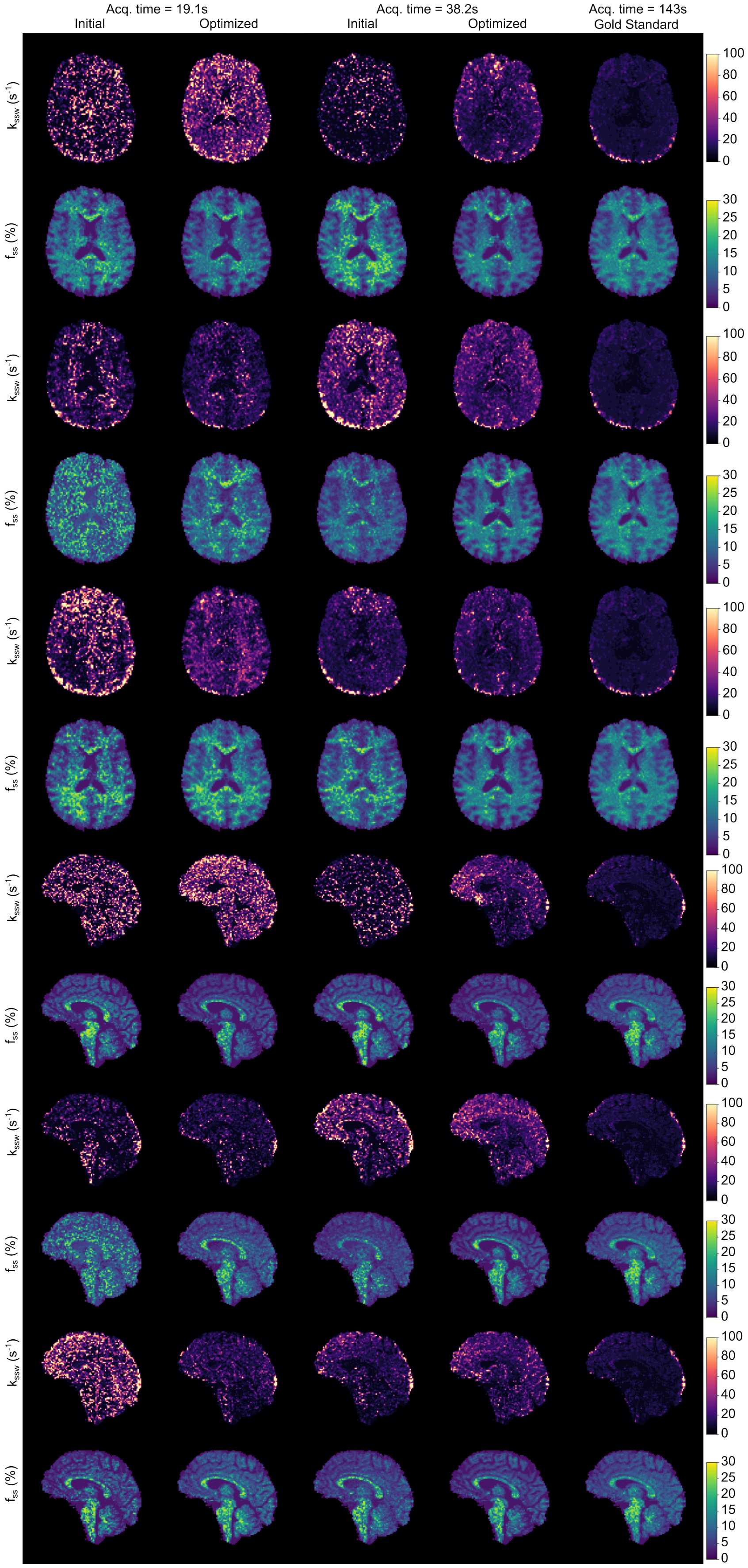

Supporting Information Figure S9. PW ST MRF imaging in healthy human volunteer #3. A representative slice is shown for each of the twelve optimization procedures performed (see Figure S3).

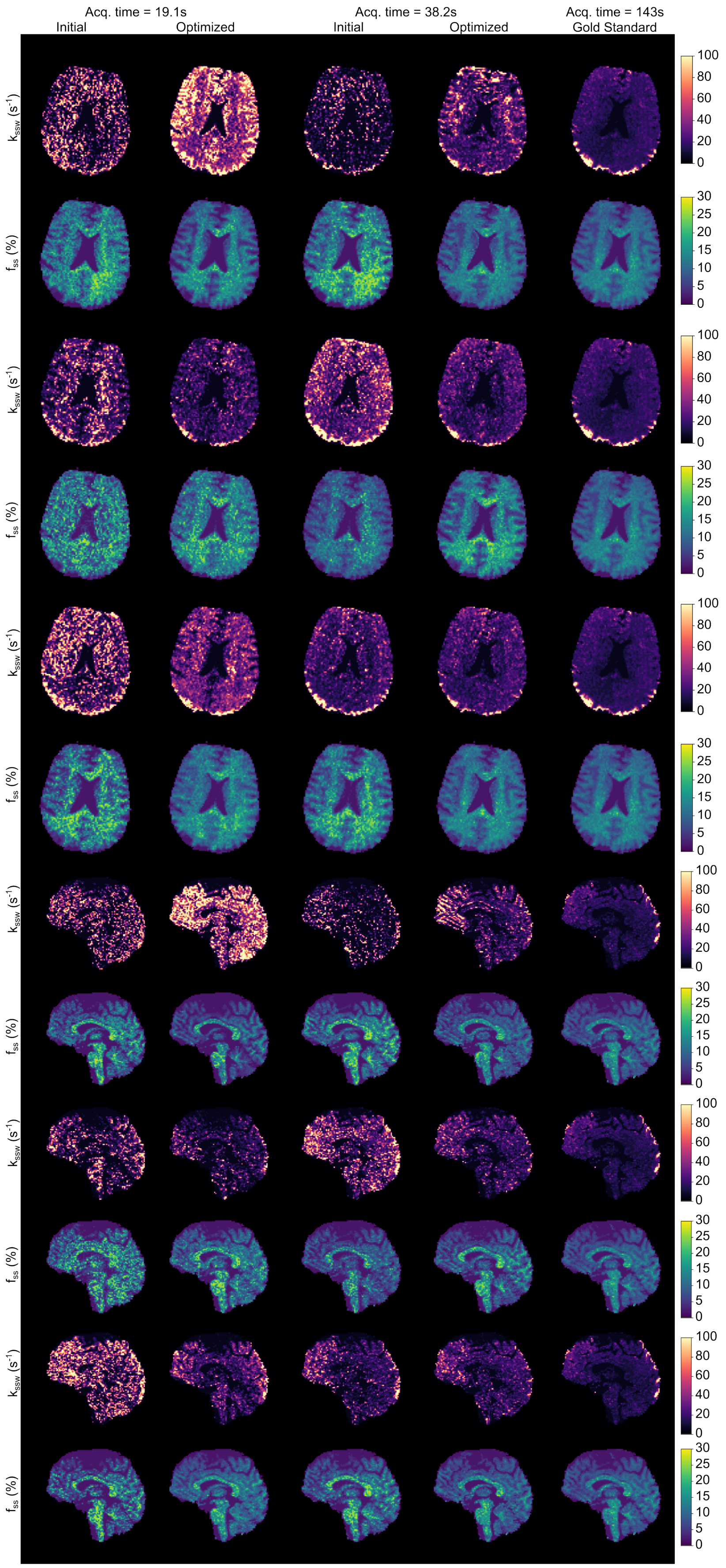

Supporting Information Figure S10. PW ST MRF imaging in healthy human volunteer #4. A representative slice is shown for each of the twelve optimization procedures performed (see Figure S3).

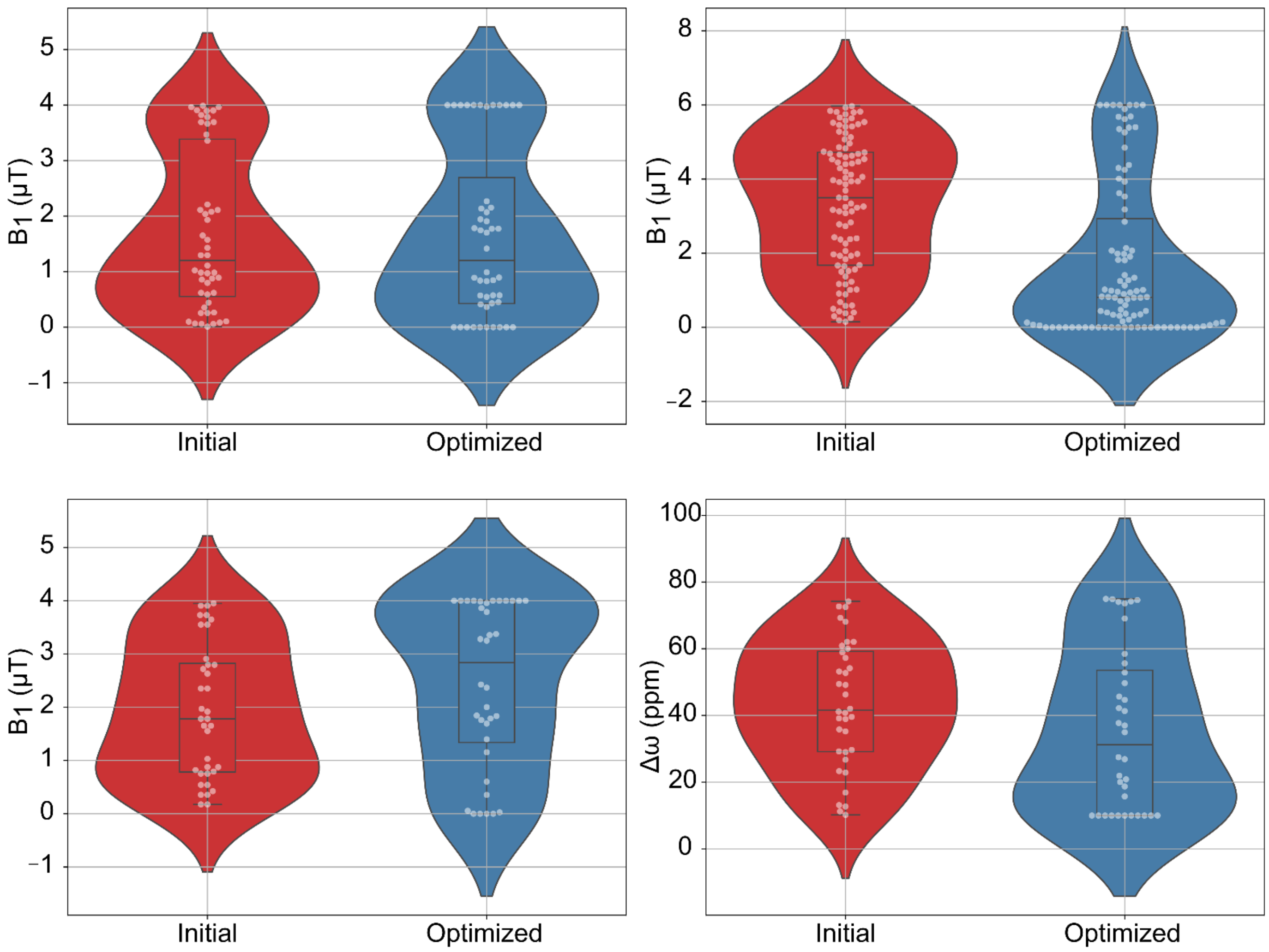

Supporting Information Figure S11. Parameter distribution of MRF acquisition protocols: A comparison between randomly initialized and CRB optimized acquisition schedules. (Top left) Distribution of saturation pulse powers used for CW phantom imaging[37]. **(Top right)** Distribution of saturation pulse powers used for PW phantom imaging[40]. **(Bottom left)** Distribution of saturation pulse powers, and **(Bottom right)** frequency offsets used for PW human imaging[40]. All other acquisition parameters are detailed in Section 2.4.

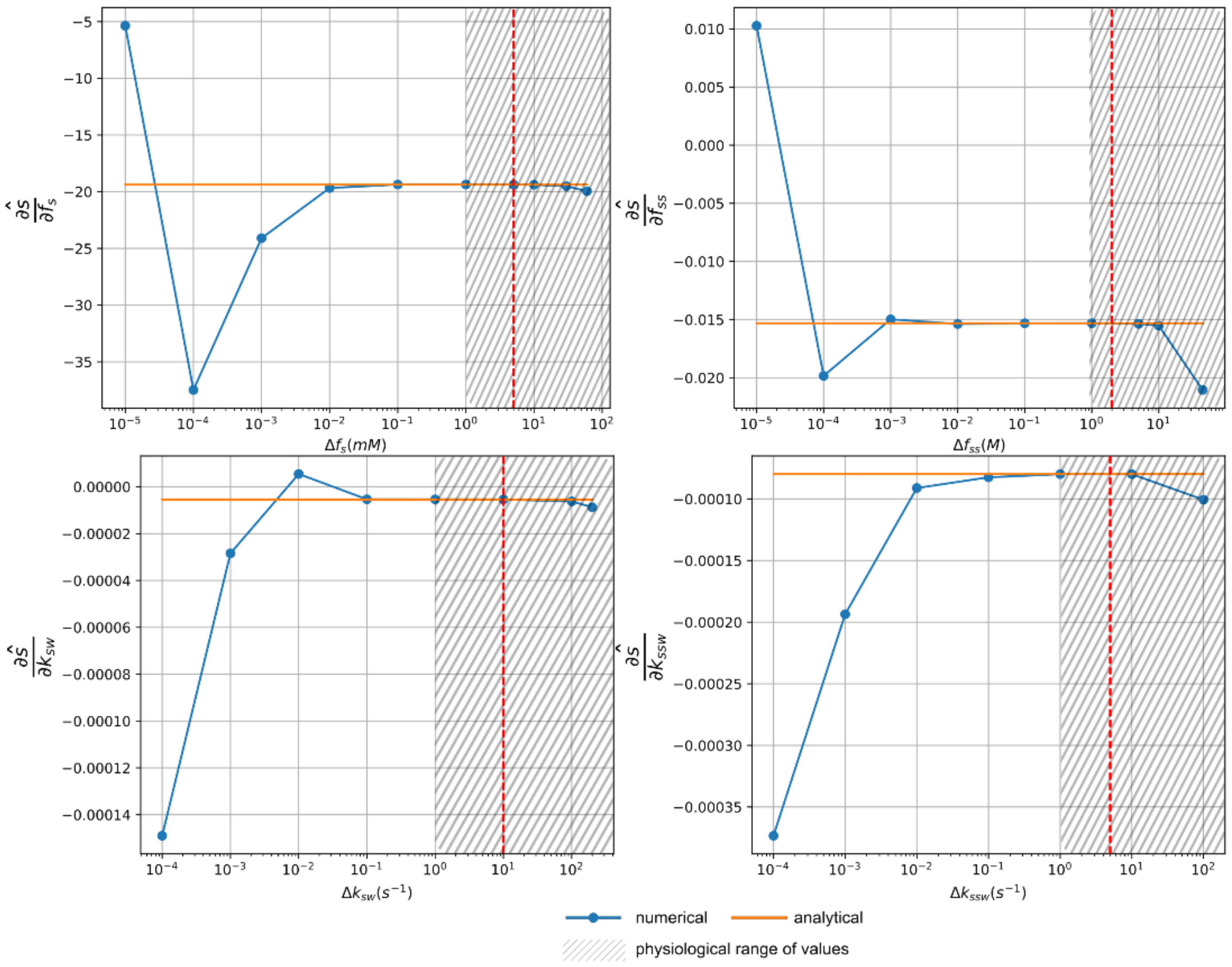

Supporting Information Figure S12. A comparison between the numerical approximation of the signal differential with respect to the quantification parameters ∂s[n;θ]/∂θ and its analytical counterparts, computed over a range of delta values for the continuous-wave saturation pulse case. Simulations were performed using a random batch of 128 samples. Note the agreement between the analytical gradient value and the selected numerical step size (red vertical dashed line).

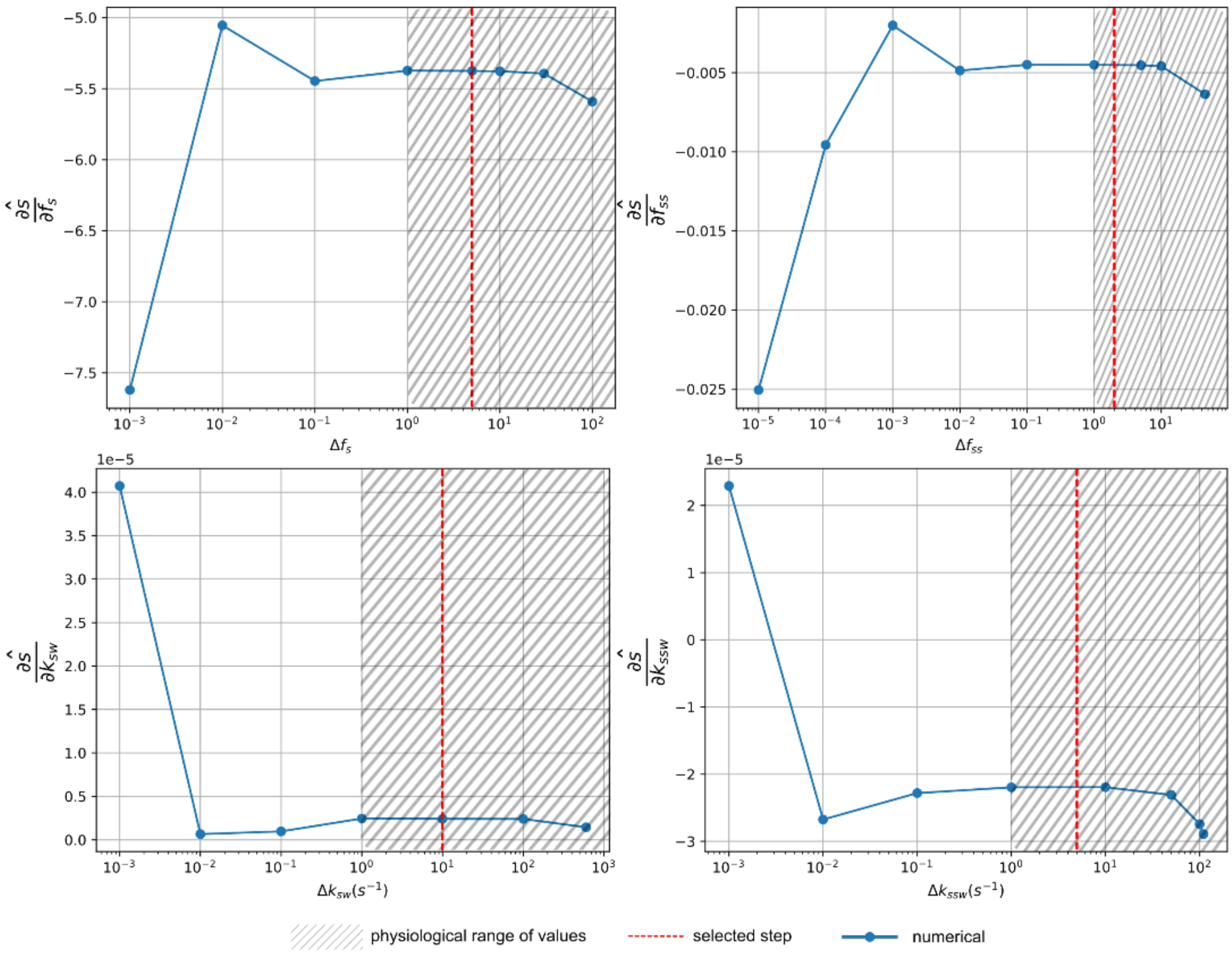

Supporting Information Figure S13. Numerical approximation for the signal differential with respect to the quantification parameters ∂s[n;θ]/∂θ computed over a range of delta values for the pulsed-wave saturation case. The simulation was performed on a random batch of 128 samples. The chosen step size (vertical red dashed line) is located in a stable gradient region.

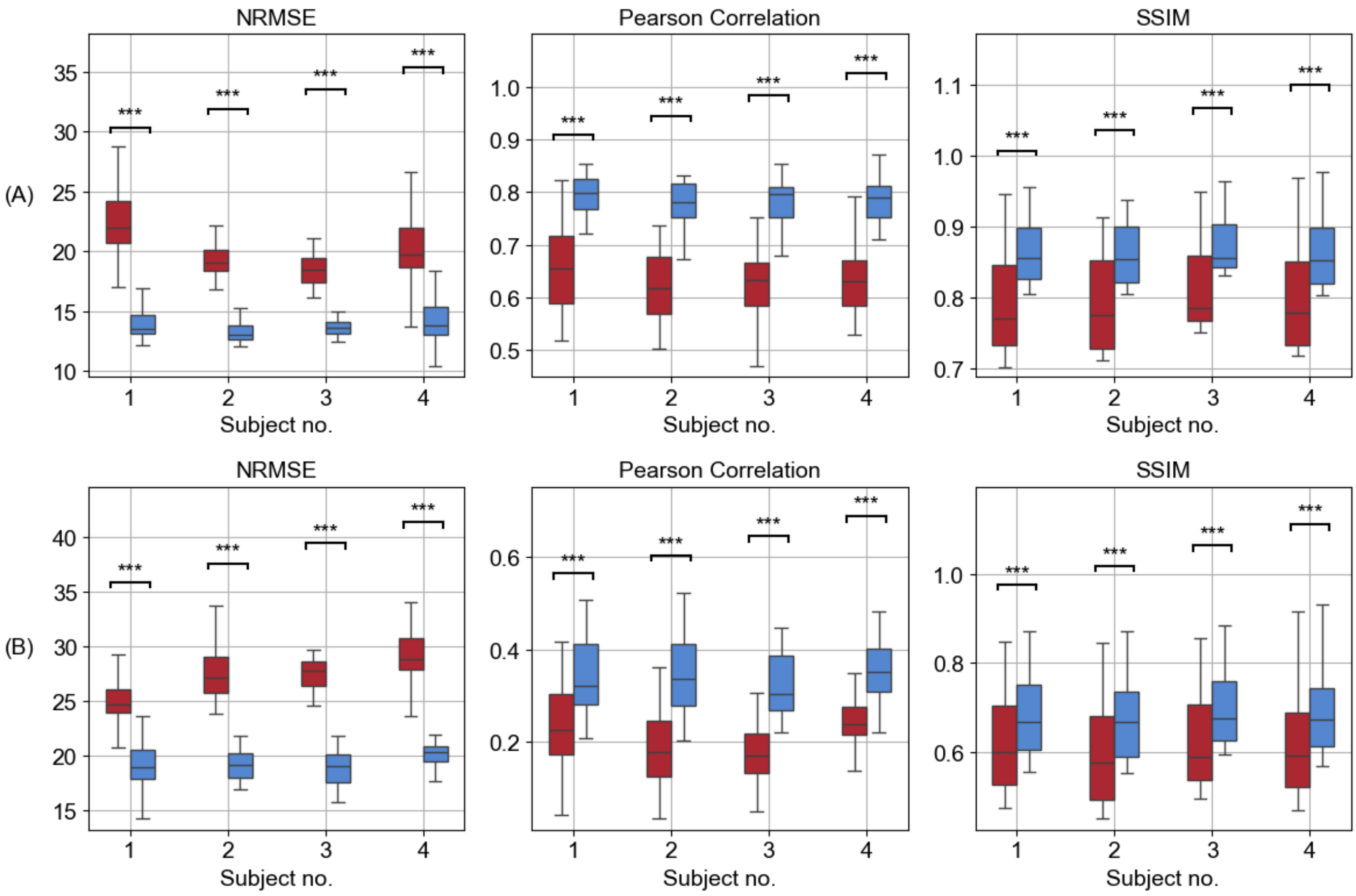

Supporting Information Figure S14. Statistical analysis of the in vivo semisolid MT proton volume fraction (A) and proton exchange rate (B) maps, following a dot-product operator that incorporated WASABI[70]-driven B_1 correction in a pixelwise manner. The NRMSE, SSIM, and Pearson's correlation values were calculated with respect to gold standard reference maps obtained using a previously established (and longer) acquisition protocol[40,56]. The red and blue box plots represent the initial and CRB-optimized acquisition protocols, respectively.

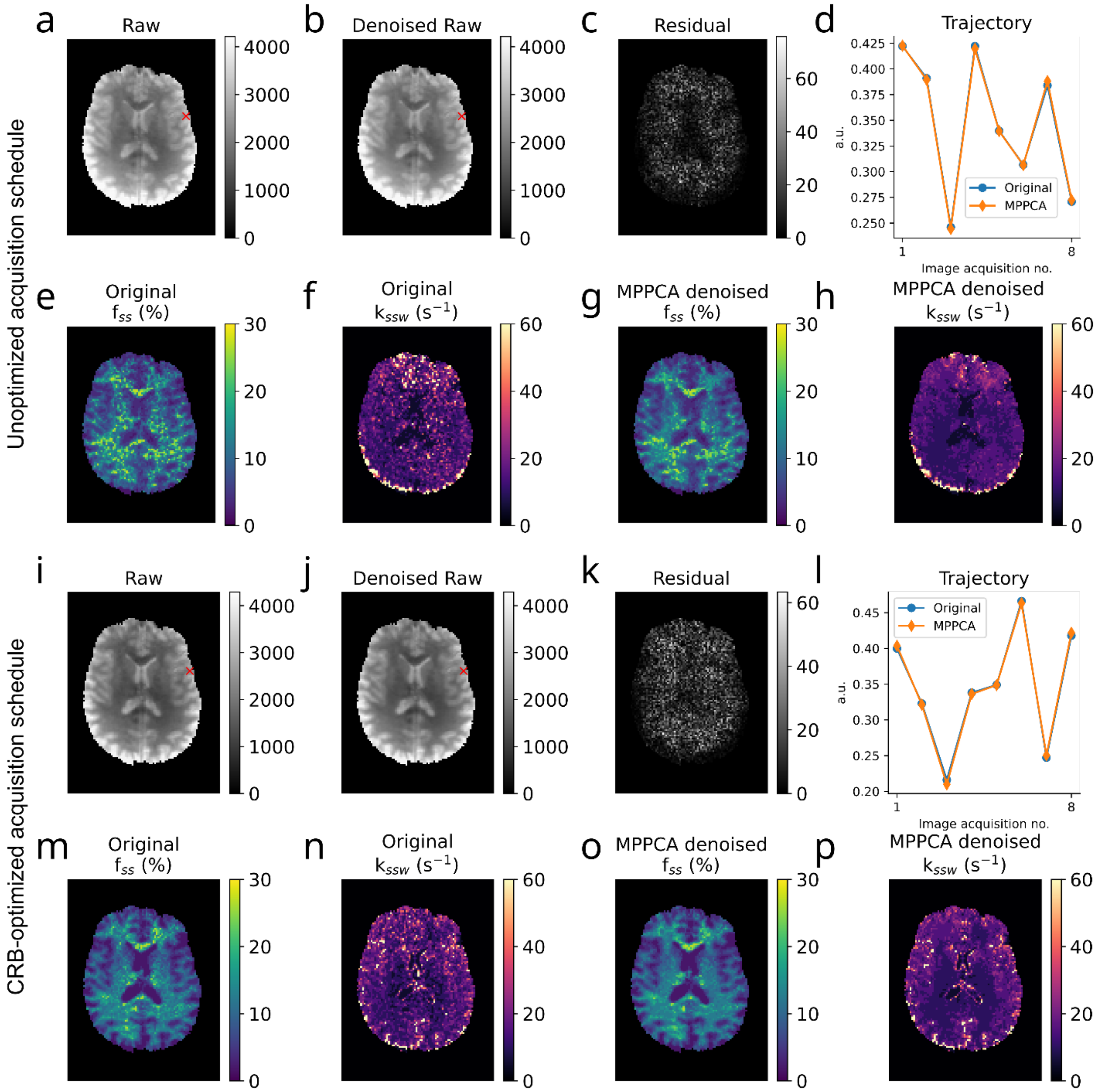

Supporting Information Figure S15. A representative example exploring the impact of de-noising the raw MRF data, for an unoptimized acquisition protocol (**a-h**), and its respective CRB-optimized counterpart (**i-p**). **a-b.** Raw MRF images before (**a**) and following (**b**) Marchenko-Pastur principal component analysis (MPPCA) denoising. **c**. The residual difference between the raw images. **d.** A comparison of the pre- and post-MPPCA signal trajectories from a random pixel shown by the red cross in **a** and **b**. **e-h**. Pre- (**e-f**) and post- (**g-h)** MPPCA quantitative semisolid MT proton volume fraction (f_{ss}) and exchange rate (k_{ssw}) maps. Note the improved SNR, despite the remaining overshoot in f_{ss} values. **(i-p)**. The same analysis, performed for the images obtained using the CRB-optimized acquisition schedule.

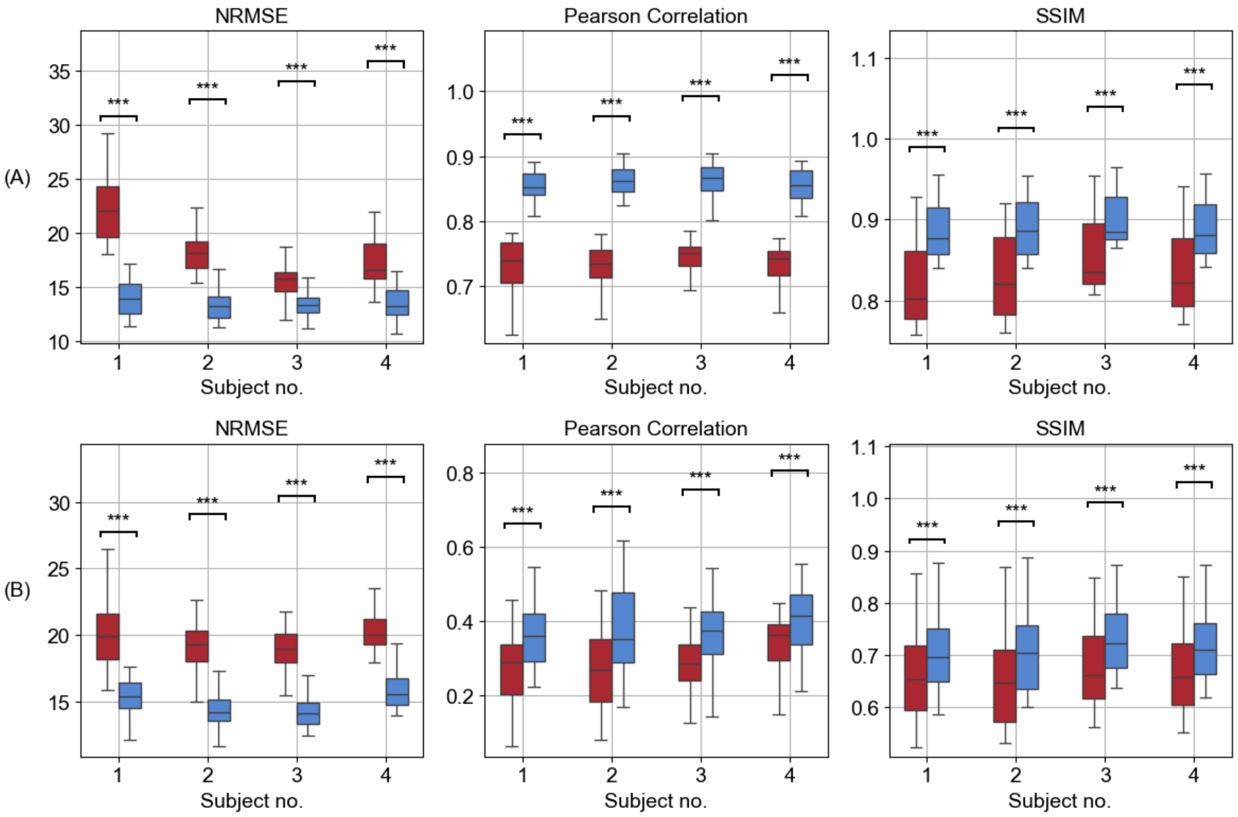

Supporting Information Figure S16. A Statistical analysis of the in vivo semisolid MT proton volume fraction (**A**) and proton exchange rate (**B**) quantification performance, following Marchenko-Pastur principal component analysis (MPPCA) denoising of the raw data. The NRMSE, SSIM, and Pearson's correlation values were calculated with respect to reference maps obtained using a previously established (and longer) acquisition protocol[40,56]. The red and blue box plots represent the initial and CRB-optimized acquisition protocols, respectively.

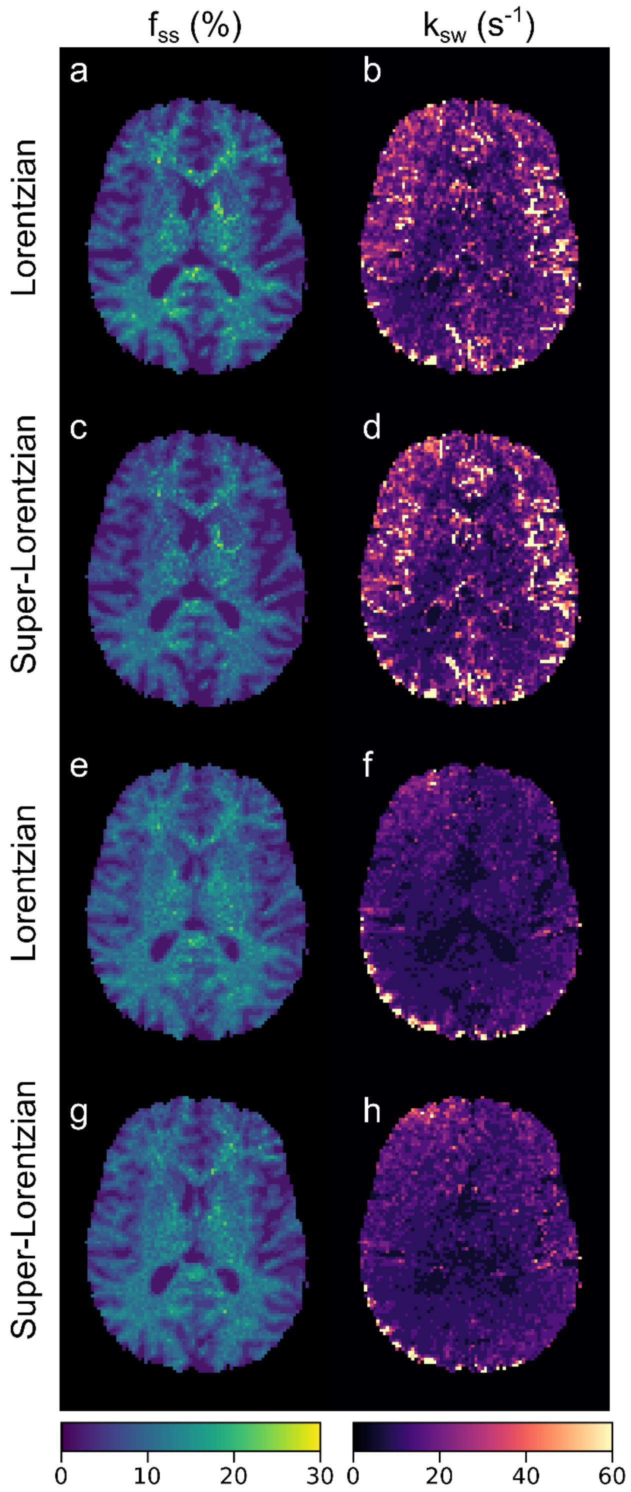

Supporting Information Figure S17. Quantitative parameter maps obtained using a CRB-optimized protocol with four raw MRF images (**a-d**) and a reference protocol employing 30 raw MRF images[40,56] (**e-h**). The images were obtained by dot-product matching to a dictionary that simulated a Lorentzian (**a,b,e,f**) or a super-Lorentzian (**c,d,g,h**) lineshape.

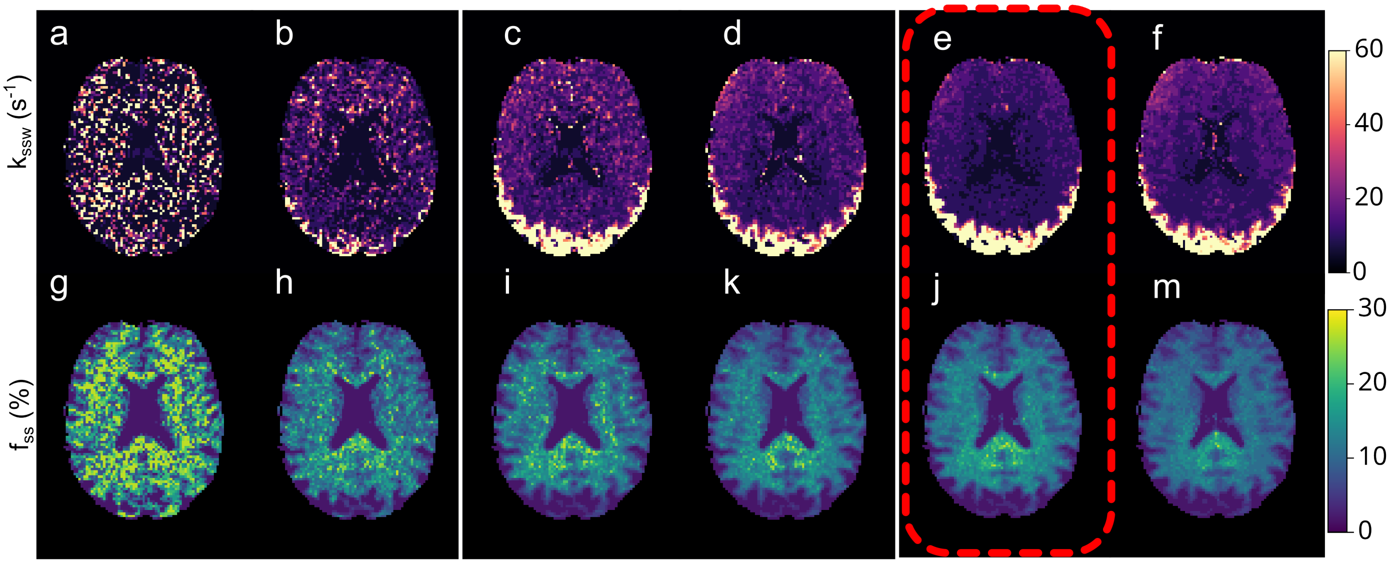

Supporting Information Figure S18. Examining the effect of super-Lorentzian incorporation on in vivo optimization. Quantitative parameter maps obtained using random protocols utilizing four (**a, g**) and eight (**c, i**) raw MRF images. The reference method[40,56] images are shown in **e, j** (marked using the red dashed square). CRB-optimization was performed after initialization with all three sequences, resulting in the parameter maps shown in **b, h, d, k, f,** and **m,** respectively. In all cases, a super-Lorentzian lineshape was used in both the optimization process and the dictionary generation.

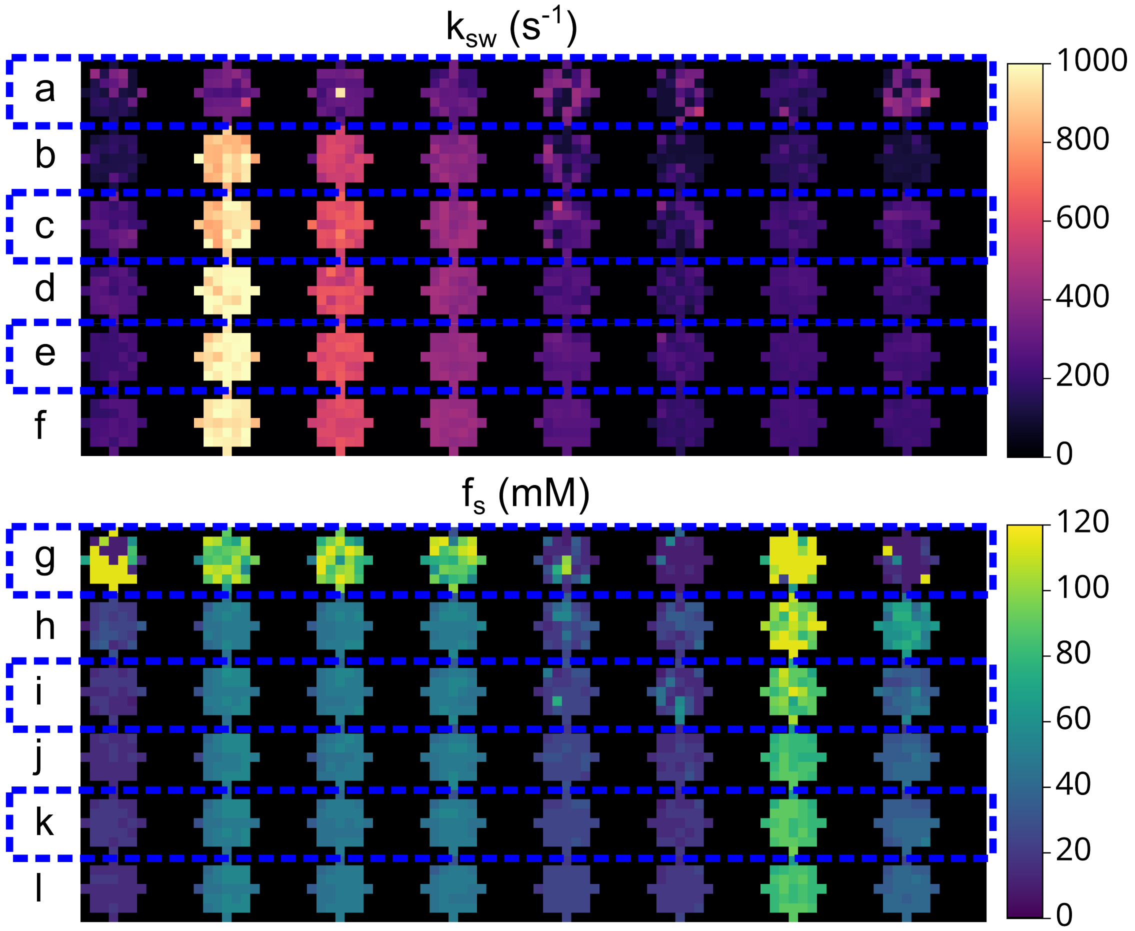

Supporting Information Figure S19. Amine proton exchange rate (top) and L-arginine concentration maps (bottom) obtained by applying a PW MRF acquisition protocol at 3T that included the first four (**a, g**), eight (**c, i**), and thirty (complete, **e, k**) acquisition parameters described in the reference protocol[40,56]. CRB-optimized protocols of length four (**b, h**), eight (**d, j**), and thirty (**f, l**) are shown for comparison. The blue dashed squares represent the quantitative parameter maps obtained using the shortened and complete reference protocol[40,56].

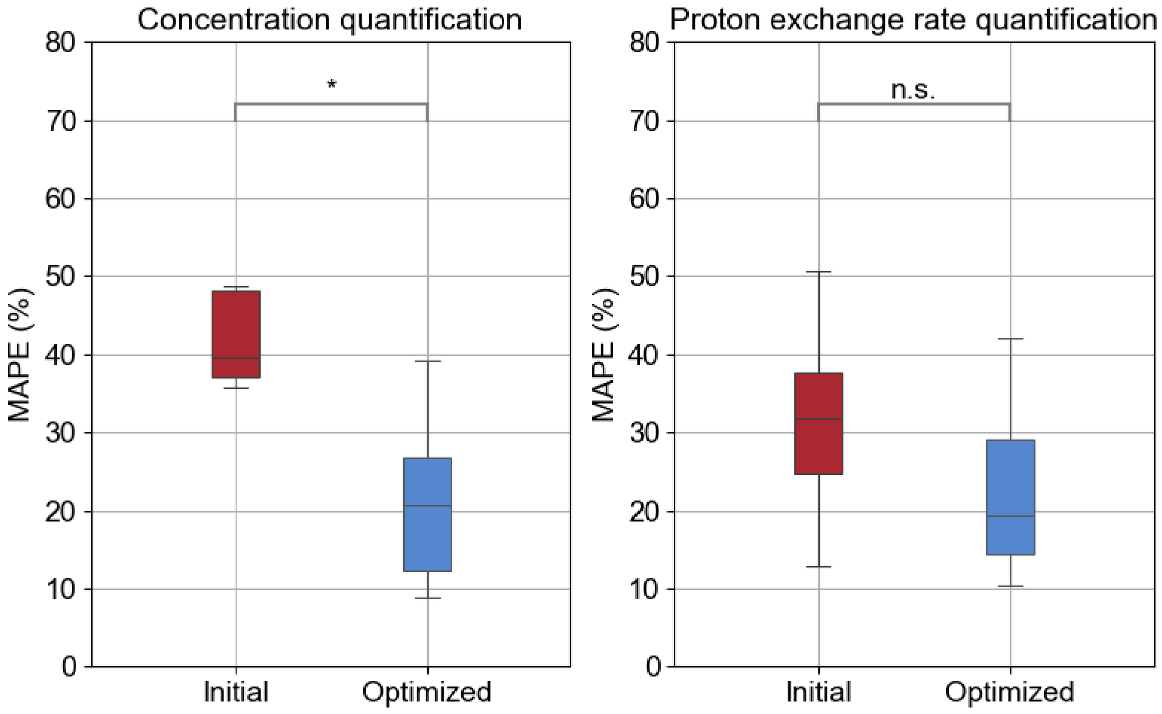

Supporting Information Figure S20. Statistical analysis of the mean absolute percent error (MAPE) across different L-arginine vials imaged using a PW MRF pulse sequence at a 3T clinical scanner. Initial sequences were based on the reference standard protocol (with various lengths)[40,56]. $^*p < 0.05$. n.s. = not significant. The red and blue box plots represent the initial and CRB-optimized acquisition protocols, respectively.

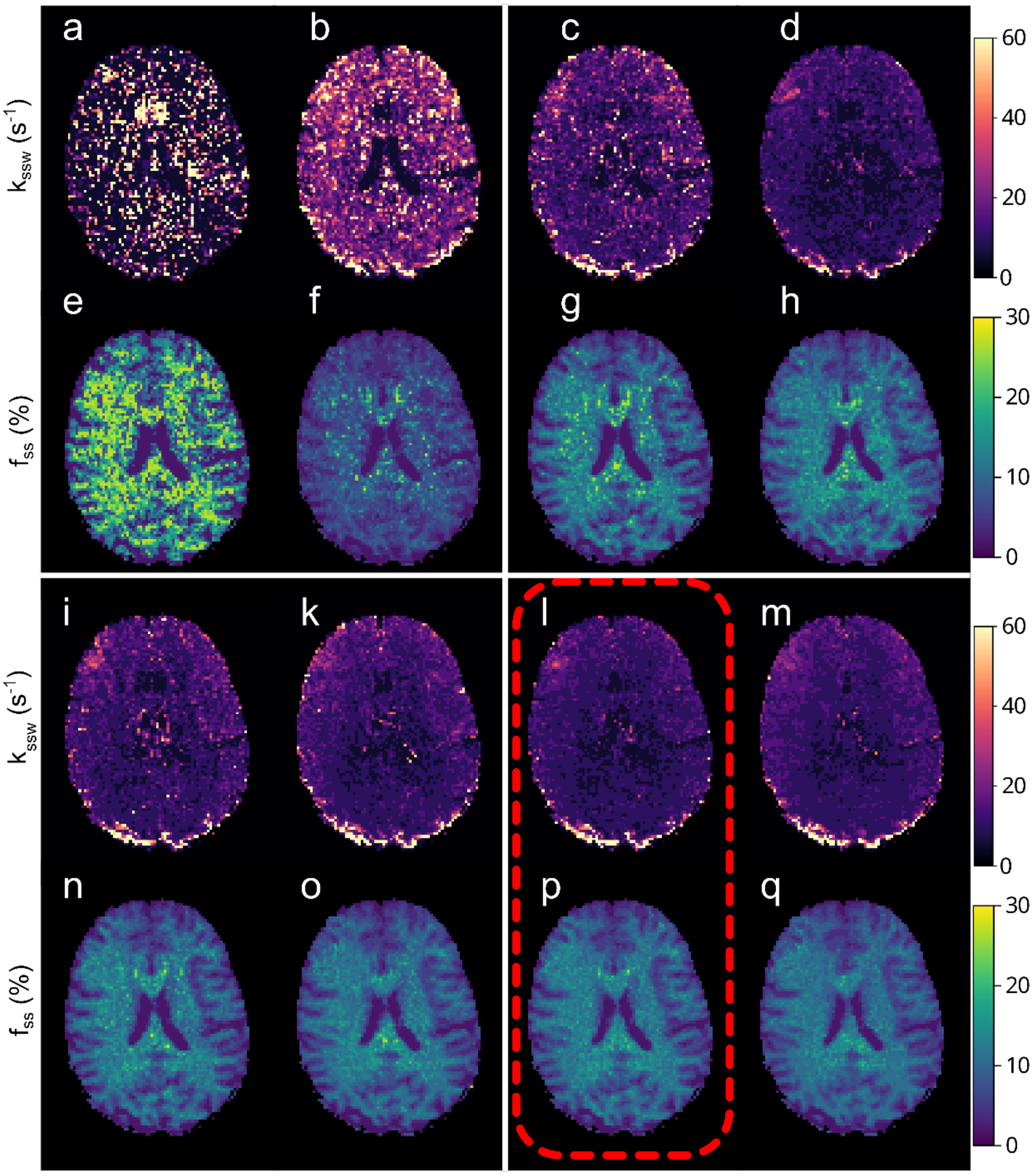

Supporting Information Figure S21. Representative quantitative parameter maps obtained using a shortened reference protocol to four (**a, e**), eight (**c, g**), and sixteen (**i, n**), raw MRF images, alongside the original (thirty raw images) reference standard[40,56] (**l, p**). The images obtained using CRB-optimized protocols (**b, f, d, h, k, o, m, q**), initialized using the shortened reference standard are also shown. The red dashed square represents the (full length) reference method quantitative maps[40,56].

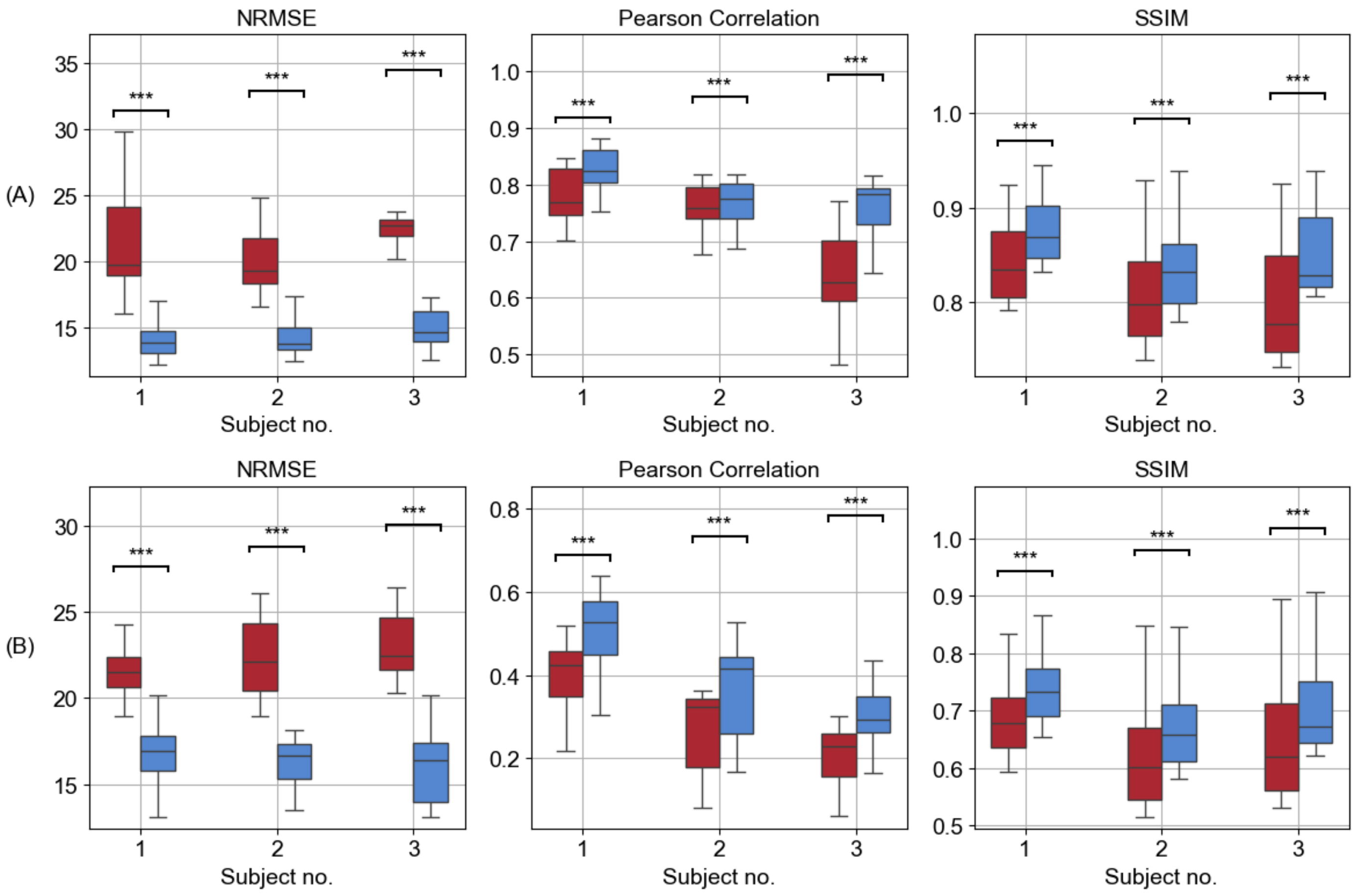

Supporting Information Figure S22. A Statistical analysis of the in vivo semisolid MT proton volume fraction (**A**) and proton exchange rate (**B**) quantification performance. The CRB-optimized potocols were initialized based on previously established reference (with various lengths)[40,56]. The NRMSE, SSIM, and Pearson's correlation values were calculated with respect to reference maps obtained using the previously established (and full length) acquisition protocol[40,56]. The red and blue box plots represent the initial and CRB-optimized acquisition protocols, respectively.

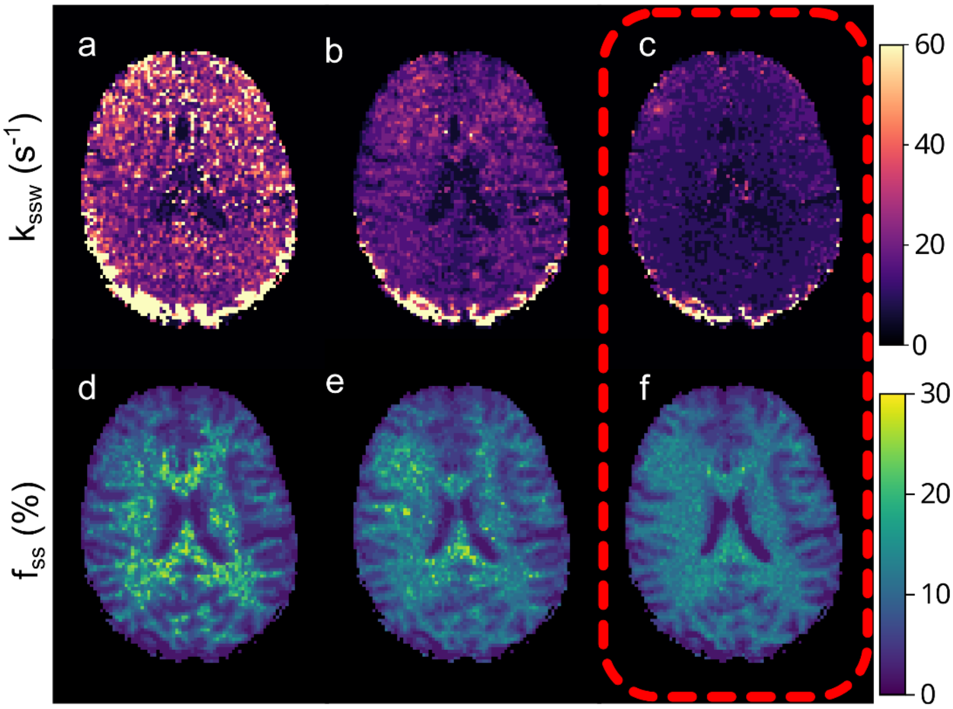

Supporting Information Figure S23. Quantitative parameter maps obtained using a (**a,d**) PW MRF sequence that utilized machine-learning based optimization[45] and (**b,e**) the CRB method following initialization using the same sequence[45]. The red dashed square (**c,f**) represents the reference standard quantitative parameter maps[56]. In all cases, the same PW saturation pulse train duty cycle, pulse duration, and 3D readout parameters were used.

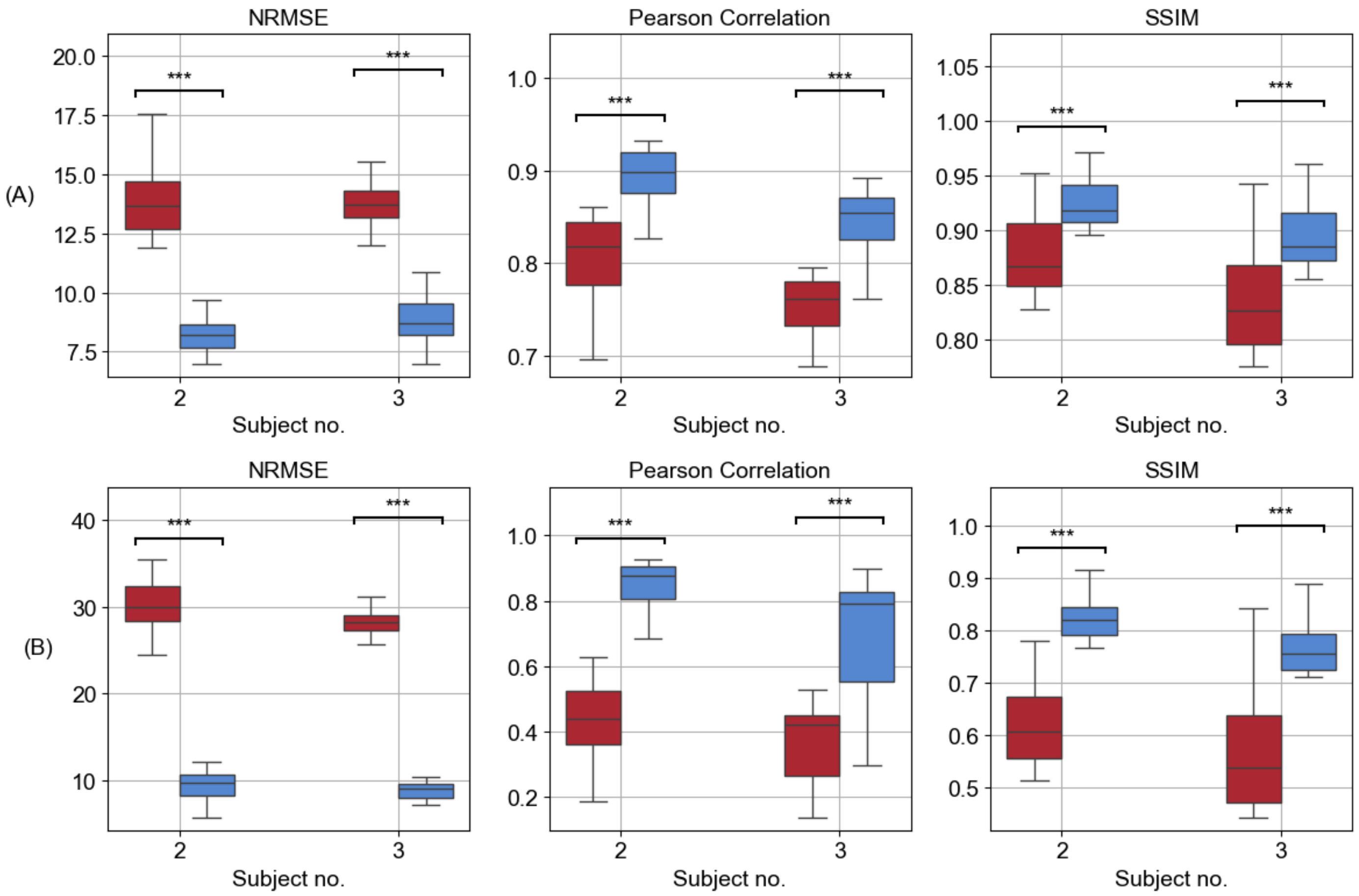

Supporting Information Figure S24. A Statistical analysis of the in vivo semisolid MT proton volume fraction (**A**) and proton exchange rate (**B**) quantification performance. CRB-optimized protocols were initialized using a previously reported protocol, which was obtained using a machine-learning based approach[45]. The NRMSE, SSIM, and Pearson's correlation values were calculated with respect to reference maps obtained using a previously established (and longer) acquisition protocol[40,56]. In all cases, the same PW saturation pulse train duty cycle, pulse duration, and 3D readout parameters were used.

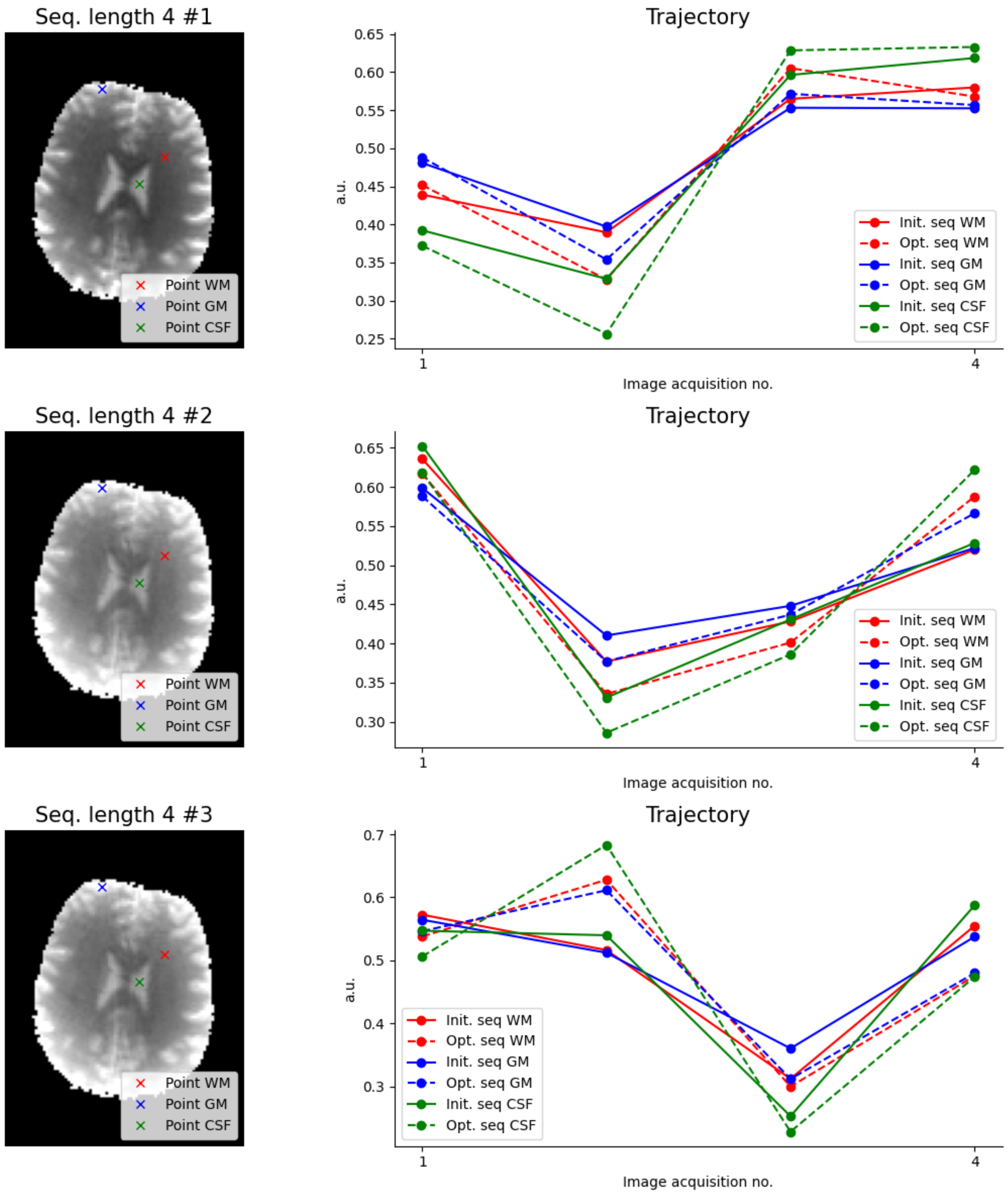

Supporting Information Figure S25. MRF signal trajectories (**right**) associated with random WM/GM/CSF pixels (**left**) for the initialized and optimized sequences (with an acquisition time of 19.1 s).

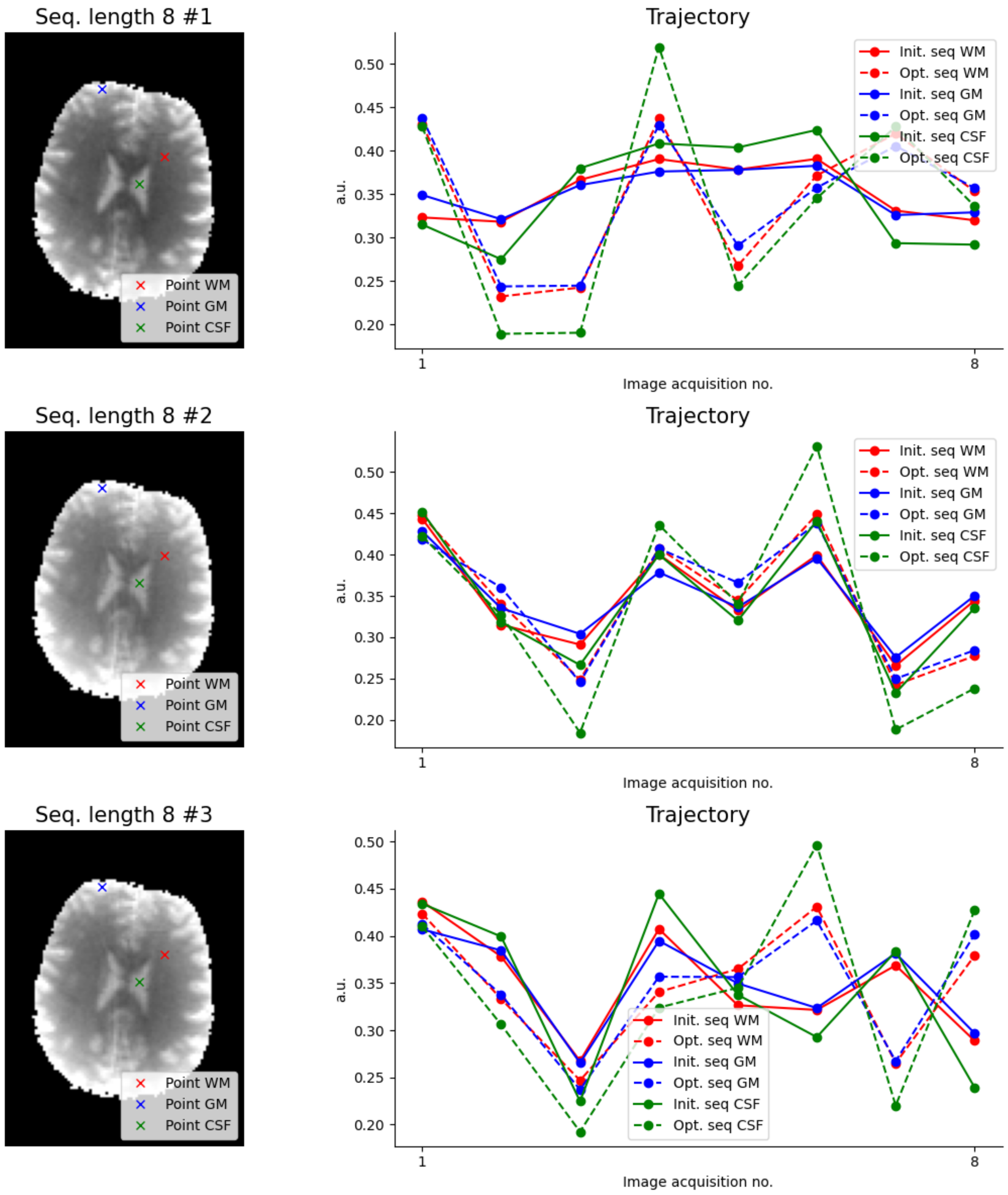

Supporting Information Figure S26. MRF signal trajectories (**right**) associated with random WM/GM/CSF pixels (**left**) for the initialized and optimized sequences (with an acquisition time of 38.2 s).

Supporting Information Table S1. Dictionary properties.

Case	7T CW L-arginine Imaging	3T PW L-arginine Imaging	3T In-vivo PW Semisolid MT Imaging
Water T1 (ms)	2500:100:3300	2500:100:3300	800:100:3500
Water T2 (ms)	600:50:1200	400:50:2000	10:10:150
T2*	-	30 ms, 10 isochromats	
Solute T1 (ms)	Equal to water T1	Equal to water T1	-
Solute T2 (ms)	40	40	
Solute concentration (mM)	10:5:120	10:5:120	
Solute exchange rate (s^{-1})	100:10:1400	100:10:1000	
Solute chemical shift (ppm)	3		
Semisolid MT T1 (ms)	-	-	1950
Semisolid MT T2 (µs)			40
Semisolid MT proton volume fraction (%)			1.8:1.8:27
Semisolid MT proton exchange rate (s^{-1})			5:5:110
Semisolid MT chemical shift (ppm)			-2
Semisolid MT Lineshape			Lorentzian
Total number of entries	665,783	792,792	129,360

Supporting Information Table S2. Computational complexity timing. In all measurements, the number of parallel workers for the dictionary generation was set to 16. An NVIDIA RTX3060 GPU was used for dot-product calculation.

	**Preclinical**[a]		**Clinical L-arg**[b]		**Clinical Brain**[c]	
Acquisition protocol length (no. raw images)	4	8	4	8	4	8
Optimization time (hours)	19.64±4.15	15.07±2.64	14.26±2.97	20.29±10.76	20.74±7.7	30.63±9.39
Dictionary generation (sec)	2.56±0.07	3.84±0.1	56.28±2.2	124.7±0.82	2.56±0.13	4.67±0.04
Dictionary generation with T2* (sec)	-		587.7±6.2	1241±13.1	22.41±1.19	46.61±2.11
GPU Dot-Product with overhead[d] (sec)	1.13±0.07	2.16±0.1	1±0.08	1.16±0.27	11.59±0.05	11.99±0.09
GPU Dot-Product (sec)	0.28±0.02	0.98±0	0.23±0	0.36±0	10.61±0.17	10.98±0.05

[a]In the CW case, a dictionary with 665,783 entries was used

[b]In the PW L-arginine case, a dictionary with 406,406 entries was used.

[c]In the clinical MT case, a dictionary with 129,360 entries was used.

[d]Initialization overhead is roughly 1 second. In all cases, timing was performed for a single slice matching, while for whole brain human imaging, it was performed for all slices.

Supporting Information Table S3. Coefficients of variance (CoV) of the estimated semisolid MT proton volume fraction (f_{ss}) and exchange rates (k_{ssw}) parameters obtained in the WM/GM ROIs of human subjects, obtained from different random protocols, full-length and shortened previously established MRF protocols[40,45,56], and their CRB-optimized counterparts.

	ROI	CoV for f_{ss}		CoV for k_{ssw}	
		Initial	Optimized	Initial	Optimized
Random initialization	Randomly initialized sequences with acquisition time = 19.1 s				
	White matter	14.31%	**8.54%**	20.85%	**20.45%**
	Gray matter	24.50%	**17.88%**	**39.19%**	55.90%
	Randomly initialized sequences with acquisition time = 38.2 s				
	White matter	13.08%	**5.72%**	29.51%	**12.85%**
	Gray matter	8.53%	**4.59%**	20.47%	**8.11%**
	All randomly initialized sequences				
	White matter	14.99%	**8.55%**	28.91%	**31.44%**
	Gray matter	23.00%	**17.44%**	**33.75%**	41.74%
Initialization based on previously reported protocols[40,45,56]	Initialization based on full length and shortened previously established protocols[40,56,40] (containing 4, 8, 10, 16, and 30 raw MRF images)				
	White matter	14.58%	**11.76%**	**30.90%**	31.34%
	Gray matter	18.30%	**12.13%**	27.23%	**20.35%**

*Values in **bold** represent improved consistency (lower CoV).
